# Impact of microbial processes on the safety of deep geological repositories for radioactive waste

**DOI:** 10.3389/fmicb.2023.1134078

**Published:** 2023-03-16

**Authors:** Miguel A. Ruiz-Fresneda, Marcos F. Martinez-Moreno, Cristina Povedano-Priego, Mar Morales-Hidalgo, Fadwa Jroundi, Mohamed L. Merroun

**Affiliations:** Faculty of Sciences, Department of Microbiology, University of Granada, Granada, Spain

**Keywords:** radioactivity, waste, deep geological repository, microorganisms, perspectives

## Abstract

To date, the increasing production of radioactive waste due to the extensive use of nuclear power is becoming a global environmental concern for society. For this reason, many countries have been considering the use of deep geological repositories (DGRs) for the safe disposal of this waste in the near future. Several DGR designs have been chemically, physically, and geologically well characterized. However, less is known about the influence of microbial processes for the safety of these disposal systems. The existence of microorganisms in many materials selected for their use as barriers for DGRs, including clay, cementitious materials, or crystalline rocks (e.g., granites), has previously been reported. The role that microbial processes could play in the metal corrosion of canisters containing radioactive waste, the transformation of clay minerals, gas production, and the mobility of the radionuclides characteristic of such residues is well known. Among the radionuclides present in radioactive waste, selenium (Se), uranium (U), and curium (Cm) are of great interest. Se and Cm are common components of the spent nuclear fuel residues, mainly as ^79^Se isotope (half-life 3.27 × 10^5^ years), ^247^Cm (half-life: 1.6 × 10^7^ years) and ^248^Cm (half-life: 3.5 × 10^6^ years) isotopes, respectively. This review presents an up-to-date overview about how microbes occurring in the surroundings of a DGR may influence their safety, with a particular focus on the radionuclide-microbial interactions. Consequently, this paper will provide an exhaustive understanding about the influence of microorganisms in the safety of planned radioactive waste repositories, which in turn might improve their implementation and efficiency.

## 1. Introduction

The risk associated to the generation and storage of radioactive waste produced by the activities of nuclear power plants is an environmental problem that must be seriously considered. It is well known that these kinds of residues contain radionuclide contaminated materials, which must be contained and managed for a long period of time until their radio toxicity decreases to natural levels. For this purpose, deep geological repositories (DGRs) have been proposed by many countries as the most immediate and safest option for their disposal. This multi-barrier system is based on the encapsulation of the radioactive waste in containers of steel, copper, titanium and nickel-based materials, which will be placed underground at depths of 500–1,000 m (IAEA, 2018; Hall et al., 2021). In addition, the containers will be surrounded by engineered (bentonite clay, cementitious materials, etc.) and natural (host rocks) barriers for their mechanical, hydraulic, and thermal protection. Indeed, clay formations will play a crucial role in many DGR designs as a host rock and engineered barriers will be used in many countries, including France, Spain, Belgium, and Switzerland. Specifically in Spain, bentonite clay from the El Cortijo de Archidona (Almería, Spain) has been selected as a reference material for the engineered barriers due to its well-characterized physical and geochemical properties (Villar et al., 2006).

A wide distribution of microorganisms has been previously reported in many materials selected to be used as barriers in the DGRs, including bentonite formations (Mijnendonckx et al., 2021; Burzan et al., 2022). Many studies have evidenced the role that many microbial processes may play in the corrosion of metal containers, on the clay mineral transformation, gas production, and the mobility of radionuclides present in radioactive waste (Beaton et al., 2019; Lopez-Fernandez et al., 2020; Hall et al., 2021). It is clear that microorganisms could threaten the safety of DGR systems. Several microbial mechanisms such as biotransformation, biosorption, biomineralization, and bioaccumulation are thought to be involved, probably affecting the radionuclide migration behavior throughout the repository. Among the radionuclides present in radioactive waste, selenium (Se), curium (Cm), and uranium (U) stand out as being of great interest. Selenium is a common component of the spent nuclear fuel present mainly as ^79^Se isotope (half-life 3.27 × 10^5^ years; Hassan et al., 2021). Although other Se isotopes such as ^75^Se (half-life 120 days) or ^80^Se (stable isotope) can be found in radioactive residues, only ^79^Se present in high-level radioactive waste is radiotoxic enough (betta particle emitter) to compromise long-term security (Abdalla et al., 2020). This element can exist in nature in different oxidation states: +VI, +IV, 0, and –II. Selenite (Se^IV^) and selenate (Se^VI^) are the most soluble and toxic forms, while elemental Se (Se^0^) and selenide (Se^-II^) are very insoluble. Cm is also a highly radiotoxic element present in nuclear spent fuel, mainly as ^247^Cm and ^248^Cm isotopes (Kooyman et al., 2018). The excellent luminescence properties of this element, as a representative of trivalent actinides (An^III^), are suitable for the study of their chemical speciation at environmentally relevant concentrations. In the same way, europium (Eu), an inactive analog of An^III^, also provides excellent luminescence properties. This inactivity makes Eu a suitable element for speciation studies of An^III^. Uranium is the main component of the spent nuclear fuel from nuclear plants constituting approximately 95% of their composition (Kumari et al., 2020). Several radioactive isotopes of uranium, such as ^238^U, ^235^U, and ^234^U, are known to have long half-lives (e.g., 4.47 × 10^9^ years for ^238^U; Mitchell et al., 2013).

This review focuses on a fundamental understanding of the microbial processes including container corrosion, gas production, clay mineral transformation, and direct interaction with radionuclides, in the safety of DGRs. Therefore, it provides useful information in predicting the microbial impact on the performance of the radioactive waste repositories. Furthermore, knowledge about these underlying mechanisms could beessential for the development of effective and accurate bioremediation strategies.

## 2. Radioactivity, nuclear energy, and radioactive waste

Radioactivity is defined as the process by which certain chemical elements emit radiation because of the spontaneous decay of their atomic nuclei. This phenomenon is characteristic of unstable isotopes, which tend to directly or progressively decay in more stable isotopes, by producing the emission of high energy radiation (Porcelli, 2018). Radioactive isotopes, also known as radionuclides, can emit 3 different types of ionizing radiation: alpha (α), beta (β), and gamma (γ). Electromagnetic waves produced by unstable nuclei are known as gamma radiation. This is the highest penetrating ionizing radiation due to the lack of mass and charge and consequently, the most dangerous to living beings. Only very thick and electron dense materials can retain them. Radioactive contamination of natural habitats may cause detrimental effects on living organisms, depending on several factors, including radiation type, received dose, affected tissue, etc. In human beings, the main radioactivity effect is based on water radiolysis, which triggers the formation of hazardous free radicals due to the ionizing action of the emitted radiation. Free radicals could lead to structural changes in biomolecules, preventing the successful fulfilling of biological functions. For example, irrevocable changes in the chemical structure of DNA would result in different kinds of tumors.

Radioactive isotopes exist naturally in soils, rocks [such as Radon-222 (^222^Rn)] and in the atmosphere (Kónya and Nagy, 2018; Porcelli, 2018). However, radioactivity is not unique to natural isotopes. Artificially produced radionuclides are much more abundant in the environment. These radioisotopes are mainly due to man-made activities, such as in medical applications in hospitals, research activities, and the nuclear power industry. One of the major global environmental risks associated to the use of these radionuclides derives from nuclear reactor accidents and nuclear waste management (Zhang et al., 2019). The large-scale release of radionuclides to the environment from nuclear activities may cause serious social and environmental problems to society (Prăvălie and Bandoc, 2018). Since 1950, approximately 20 nuclear accidents have occurred in the world (Burns, 2012; Lelieveld et al., 2012), being that of Chernobyl on 26 April 1986, undoubtedly the biggest nuclear disaster ever known. To date, this catastrophic event has contributed to the highest release of radioactive material to the environment (Burns, 2012; UNSCEAR, 2013). Cesium-137 (^137^Cs) and Iodine-131 (^131^I) are examples of some of the most dangerous radionuclides emitted during that episode. Not all radionuclides have a deep impact on our lives, but some of them are especially hazardous due to their half-life, type of radiation emitted, concentration, etc. The study of trivalent actinides (An^III^) such as curium (^247^Cm, ^248^Cm) or americium (^242^Am), other actinides such as neptunium (^237^Np) or uranium (^235^U), and some significant radioisotopes of selenium (Se), is extremely important due to their high radiotoxicity (Hamed et al., 2017; Kooyman et al., 2018). Among all the An^III^, curium (Cm) is one of the most studied. Cm is a very toxic radionuclide due to the α-activity of some isotopes, such as ^247^Cm and ^248^Cm, characteristic of spent nuclear fuel (Gorietti et al., 2017; Kooyman et al., 2018). Many of the studies focused on the study of Cm use europium (Eu) as an inactive analogous of An^III^. This inactivity, together with its excellent luminescent properties, makes Eu an exceptional element for the study of An^III^ (Ansoborlo et al., 2007). Most of the Se radioisotopes have a short half-life ranging from 20 s, in the case of ^77m^Se, to 120 days, in the case of ^75^Se. The only one of special interest due to its high radioactivity and hazard to the environment and the long-term safety of future DGR, is ^79^Se (Atwood, 2010). The radioisotope ^79^Se presents a half-life of about 3.27 × 10^5^ years and is mainly produced in the nuclear industry through fission reactions of ^235^U and other radionuclides such as ^239^Pu. As an artificial radionuclide, the only possible source of ^79^Se in nature would be nuclear accidents or the release of nuclear waste to the environment.

The high global energy demand is considerably increasing nowadays, mainly due to demographic and industrial growth. Almost the total global energy is still supplied by non-renewable sources such as fossil fuels and resources. This fact makes the development of renewable energies or new alternatives such as the nuclear power crucial. Despite the danger of nuclear reactors present, this low-carbon technology could be a great opportunity to minimize the consequences of global warming, one of the toughest environmental problems facing human society today (IAEA, 2015). Obviously, simultaneous strategies based on the establishment of renewable energies are crucial for this purpose. Despite the above mentioned comments, there is a continuous controversy about the use of nuclear energy due to the associated risk involved, such as environmental pollution and the generation and management of radioactive waste. The proper and safe storage of the nuclear residues produced implies a real threat to the environment because a completely safe solution is still non-existent. The IAEA, which is officially in charge of radioactive waste management, classify this in six different types (IAEA, 2009a):

- Exempt waste (EW): contains very low concentration of radionuclides, resulting in their exclusion from the regulatory control of radiation protection.
- Very short-lived waste (VSLW): contains very short half-life radionuclides that must be stored until the end of their radioactivity. It is mainly produced in hospitals and research centers.
- Very low-level waste (VLLW): waste containing a radioisotope concentration slightly higher than EW, which can be disposed in near surface landfill type facilities with limited regulatory control. These residues usually include natural radionuclides from soil, rocks, or the mining industry.
- Low level waste (LLW): includes limited amounts of long-lived radioisotopes, which requires isolation for periods of up to a few hundred years in near surface disposal.
- Intermediate level waste (ILW): waste including long-lived radionuclides in significant quantities to be stored in higher security levels than those provided by near surface facilities. Therefore, this type of residue requires to be isolated at greater depths ranging between tens and hundreds of meters.
- High level waste (HLW): contains large amounts of long-lived radionuclides and requires a greater degree of containment and isolation than ILW to guarantee long-term security. This type of residue is mainly composed of nuclear waste, which is the most hazardous one because of its radioactivity, which may persist for up to 1,000 years (Horvath and Rachlew, 2016). HLW generates significant quantities of heat as a result of the atomic nuclei decay of radionuclides. For this reason, heat dissipation is an important issue to be considered during the design of a repository for this kind of waste. Nowadays, the production of HLW is extremely alarming, since it is estimated that each nuclear reactor (approximately 440 operational worldwide) produces around 30 tons per year (Gerstner, 2009; IAEA, 2009b; Rosa et al., 2010; Alwaeli and Mannheim, 2022).

The high generation rate of radioactive waste is a major environmental concern that needs to be adequately solved as soon as possible. Reprocessing spent nuclear waste is an example to partially solve this problem. According to the IAEA, more than 5,000 tons are reprocessed every year (IAEA, 2009b; Handrlica, 2019). However, it seems evident that this procedure is not enough to reduce the total volume of radioactive waste in the world. In the last few years, the global scientific and political consensus has determined the isolation of radioactive waste in deep geological repositories (DGRs) as being the safest option for its long-term management (NEA, 2020a).

## 3. Deep geological repository systems

**Figure 1:**
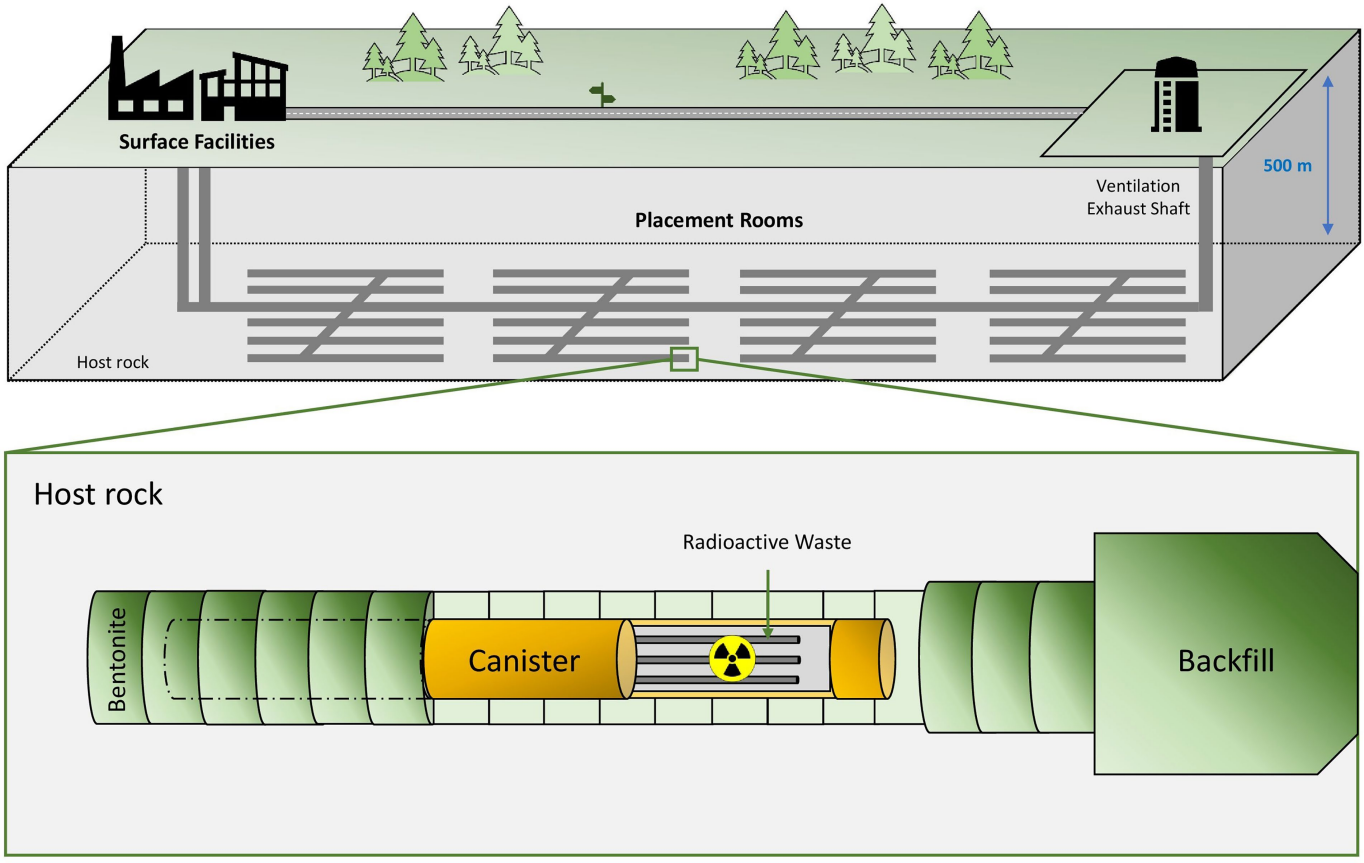
Diagram of the deep geological repository system according to the Nuclear Waste Management Organization (NWMO).

The deep geological repository (DGR) concept was introduced in 1995 by the Nuclear Energy Agency (NEA) with the aim to safely isolate radioactive waste for long-term periods (NEA, 2020b). The DGR system is based on the disposal of radioactive waste at a depth of around 1,000 m underground (Figure 1). Multi-barrier systems composed of natural (soil, host rock, etc.) and artificial barriers such as metal canisters and filling/sealing materials are planned to properly isolate the residues (Ma et al., 2018). Clay, crystalline rock and salt deposits have been studied and selected as the best options for natural barriers due to their excellent physico-chemical properties (Ahonen et al., 2016). France, Belgium, and Switzerland have chosen clay formations as the preferred host rock option for their repositories, while crystalline rock (granite) has been selected by other countries such as Finland, Sweden, Canada, and the Czech Republic (Ahonen et al., 2016). However, the EEUU is considering salt deposits for their geological repository emplacements. Regarding the artificially engineered barriers, copper, steel, and concrete are planned to be used as canister materials, while cementitious materials and bentonite clay formations seem to have the best sealing capabilities. It is worth noting that all these materials would act as a protective shield against radiation (Abrahamsen et al., 2015). In these systems, an initial period with oxic conditions is expected mainly for the introduction of oxygen accumulated during the construction of the disposal facility. However, after the closure of the repository, anoxic and reducing conditions will prevail because of the low oxygen levels underground, the presence of specific electron donors and acceptors, and the dilution of reduced minerals (Duro et al., 2014). For all the above-mentioned reasons, DGR systems provide a stable environment, making them the best solution for the long-term isolation of long-lived radionuclides characterizing HLW.

Nowadays, several countries are planning or have already started the construction of their own DGRs (Nuclear Waste Management Organization, 2022). For example, The Netherlands, Ukraine, or Spain are currently investigating the type of geology to be used (crystalline rocks, clays or salt deposits). The United States and South Korea governments have agreed to build a DGR facility, but they are still in the early planning stages. The *Nuclear Waste Management Organization* (NWMO) of Canada is currently studying about the crystalline and sedimentary site selection both in the province of Ontario. Similarly, the *Nuclear Waste Management Organization of Japan* (NUMO) has an active program for the site selection. Further, China has announced the pre-selection of a national plan for the storage of radioactive waste in granite geological areas in the Gansu province. Switzerland has also already selected the location of its DGR (north of Zürich), but still expects to submit a license application by 2026. France, one of the biggest nuclear energy producers, is designing through the *Agence Nationale pour la gestión des Déchets Radioactifs* (ANDRA), a DGR called Cigéo over a clay rock emplacement to the east of the country. License applications are still being prepared for Cigéo and its construction is programmed to begin as early as 2023. Similarly, the Swedish government has recently granted permission to the SKB company to build a deep disposal approximately around the mid-2020s with final disposal structure expected to be finished around 2030. The most advanced country in this respect is Finland, where the Onkalo Facility is expected to begin operating in 2025. The Onkalo DGR will be the first in the world to start the final disposal of spent nuclear fuel. The objective is to store HLW and ILW using a system based on cementitious materials for both containers and sealing materials.

## 4. Diversity and influence of microorganisms in DGR systems

Microorganisms are widely described for their ability to colonize almost every single habitat on earth, and DGRs are no exception. The high diversity of microorganisms in geological formations selected for their optimal use in the disposal of radioactive waste such as clay, granite, and saline deposits, have been reported (Bachran et al., 2018; Lopez-Fernandez et al., 2018a, 2021). Many studies not only about microbial diversity, but also the activity and influence of biochemical processes on the safety of these systems are currently on-going. In those countries where clay has been chosen for their respective DGRs, there have been recent studies. This is the case of Opalinus clay in Switzerland, Boom clay in Belgium, Cavallo-Oxfordian in France, and bentonite clay in Spain (Mijnendonckx et al., 2021; Povedano-Priego et al., 2021; Burzan et al., 2022; Shrestha et al., 2022). Bentonite clay formations have been described as a reference material for their use as artificial barriers in DGRs due to their excellent mineralogic, physic-chemical, mechanistic, geochemical, and hydraulic properties (Villar et al., 2006). Specifically, they present low permeability (decrease in groundwater filtrations), mechanical support (provide stability against container weight), high ion exchange capacity (radionuclide retention), high plasticity, swelling capacity (self-sealing of cracks), thermal conductivity, and optimal compaction properties (García-Romero et al., 2019). The results obtained by Lopez-Fernandez et al. (2014, 2015, 2018b) revealed the high microbial diversity in bentonite formations from the El Cortijo de Archidona (Almería, Spain). Most of the species identified correspond to facultative and obligate aerobic microorganisms. Some of them have been previously described for their ability to interact with radionuclides and heavy metals characteristic of radioactive waste. This is the case of bacterial genera such as *Acidovorax, Variovorax, Pseudomonas, Stenotrophomonas*, and *Ralstonia* (Choudhary and Sar, 2009; Hedrich et al., 2011; Hupert-Kocurek et al., 2013; Gerber et al., 2016; Ruiz Fresneda et al., 2018; Ruiz-Fresneda et al., 2020a). Indeed, the bentonite-isolate bacterium *Stenotrophomonas bentonitica*, exhibits a great versatility and capacity to interact with different elements such as Se^IV^, Cm^III^, or U^VI^ through different microbial mechanisms (Lopez-Fernandez et al., 2014; Ruiz Fresneda et al., 2018, 2019, 2020b). Opalinus clay from the Mont Terri rock laboratory (Canton Jura, Switzerland) also shows the presence of relevant microbial activity. Although there are limited conditions for microbial survival in Opalinus clay, a rich and diverse group of microorganisms seem to be ubiquitous. This suggests that many microorganisms could have been introduced mainly as a result of anthropogenic activities including construction, operational activities and experimental installations and also due to natural geological processes such as water infiltration, landslides, fissures, etc. Specifically, sulfate-reducing bacteria (*Desulfosporosinus*, *Desulfotomaculum, Desulfocapsa*), nitrate- and nitrite-reducing bacteria (*Pseudomonas*, *Thiobacillus, Acidovorax*) and other bacterial genera (*Pleomorphomonas*, *Peptococcaceae*, *Sphingomonas*) have been found (Leupin et al., 2017). Another clay type that has been investigated in depth is Boom clay, considered as a potential host formation in Belgium. Mijnendonckx et al. (2019, 2022) detected the presence of methanogenic Archaea, such as *Methanobacterium alcaliphilum* and *Methanomassiliicoccus luminyensis*, nitrate-reducing bacteria (*Acidovorax*, *Simplicispira*, *Hydrogenophaga*), or sulfate reducing bacteria (*Pseudomonas*), among others.

To summarize, most certainly the DGR systems will not be sterile environments. Not only will indigenous microorganisms from soils, barriers and different materials be present, but also allochthonous microbes introduced accidentally during the construction of the repositories. Although the conditions will not be the best for microbial growth, it should be emphasized that the presence of nutrients, electron donors and acceptors, carbon and nitrogen sources will help the development and metabolic activity of the microorganisms. Even in the presence of ionizing radiation, some microorganisms from sediments have been described to be able to tolerate dose rates representative of gamma radiation emitted from radioactive waste (Brown et al., 2015). Not only microorganisms, but also components of these sediments such as Fe^III^, NO_3_^-^, or SO_4_^2-^ can remain active. Anyway, some microorganisms can immobilize radioactive isotopes through passive processes (biosorption, bioaccumulation, etc.), even if they do not survive radiation (see section 4.4).

Sulfate-reducing, nitrate-reducing, methanogens, iron-reducing, metal-resistant, and other microorganisms will potentially affect the environment and hence the safety of the DGRs through different processes including gas production, metal corrosion, modification of the redox conditions, mineral clay transformation, and radionuclide interaction. For all the above-mentioned reasons, microorganisms must be considered when evaluating the security level of DGRs.

### 4.1. Corrosion of the metal canisters

Bacteria can adhere to the surface of different inorganic matrices through the production of extracellular polymeric substances (EPSs) promoting bio-corrosion, known as microbially influenced corrosion (MIC) of materials such as steel, copper, concrete, etc. (Kip and Van Veen, 2015). MIC could be mediated by the direct or indirect action of electrochemical reactions conducted by microorganisms (Videla and Herrera, 2009). Within the concept of the DGRs, the oxygen will be gradually consumed after their closure, so an anoxic environment will be predominant (Rashwan et al., 2022). Under these DGR conditions, sulfate-reducing bacteria (SRB) could obtain energy through the reduction of sulfates or other oxidized inorganic sulfur compounds, resulting in sulfide production (H_2_S). This is one of the most important biogenic corrosive compounds in the DGR environment and it accelerates the corrosion rate of metal canisters (Bagnoud et al., 2016a).

**Figure 2:**
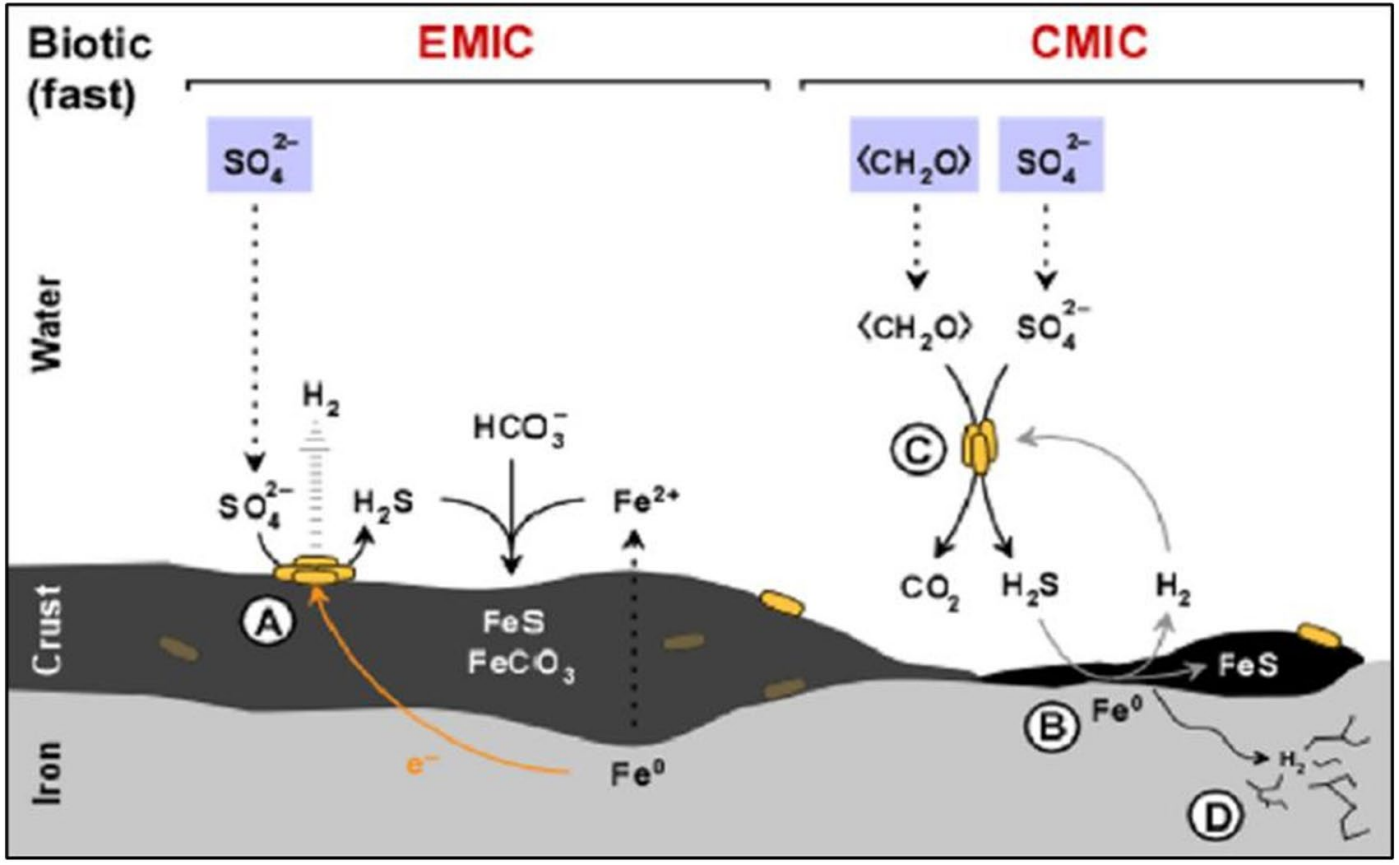
Diagram of the influence of sulfate-reducing bacteria (SRB) activity in the corrosion of iron. **(A)** Electrical microbially influenced corrosion (EMIC) mechanism. **(B)** Chemical microbially influenced corrosion (CMIC) mechanism. **(C)** Overall representation of CMIC. **(D)** Sulfide stress cracking. Modified from Enning and Garrelfs (2014).

The nature of the metal canister to be used depends on the DGR concept of each country. Copper (e.g., Canada, Finland, and Sweden) and steel-based materials (e.g., Belgium and the Czech Republic) are the predominant options for the canisters. Regarding steel-based material, Enning and Garrelfs (2014) reported the SRB contribution to the corrosion of this material through two main mechanisms: electrical microbially influenced corrosion (EMIC), and chemical microbially induced corrosion (CMIC; Figure 2). EMIC is a direct mechanism where SRB are involved in the speed up of abiotic corrosion. In this process, specially adapted SRB pull out electrons from elemental iron leading to the disposition by means of electro-conductive iron sulfides and releasing excess electron acceptors such as H_2_ (Figure 2A). On the other hand, CMIC is the indirect mechanism that results from the sulfidogenic degradation of organic matter under oxygen-free environments resulting in the production of biogenic and corrosive sulfide which reacts with the metallic iron (Figures 2B,C). Furthermore, the biogenic formed hydrogen sulfide can cause sulfide stress cracking of the iron (Figure 2D). FeS may, temporarily, act as a protective action against iron corrosion as it is strongly adhered to the metal surface through the direct reaction of the dissolved sulfide with metallic iron (Newman et al., 1992; Sun and Nešic, 2007). Several studies have reported MIC on steel-based materials by the activity of SRB. Xu et al. (2008) detected the presence of kansite (Fe_9_S_8_) and pitting corrosion in stainless steel in the presence of *Desulfovibrio* sp. and *Leptothrix* sp. El Hajj et al. (2010) found mackinawite (FeS_x_) on P235H steel mediated by the activity of extremophiles and spore-forming SRB such as *Desulfosporosinus* sp., *Desulfotomaculum* sp., and *Thermosubterraneum* sp. The generation of biogenic mackinawite was also reported by Černoušek et al. (2019) when carbon steel MIC with *Desulfomicrobium* sp. and *Desulvibrio* spp. was studied. It should be noted that, although this review focuses on the SRB group, other bacterial communities can promote the steel-based material corrosion processes. Shrestha et al. (2021) highlighted the key role of nitrate-reducing bacteria (NRB), such as *Methyloversatilis*, *Pseudomonas* and *Brevundimonas,* related to carbon steel corrosion since they could be involved in the formation of corrosion compounds such as magenetite, mackinawite [FeS_(1 − x)_], akageneite [Fe^3+^O(OH, Cl)], and rozenite [Fe^2+^SO_4_·4(H_2_O)]. All these authors concluded that the biofilm formation promotes and accelerates the corrosion.

**Figure 3:**
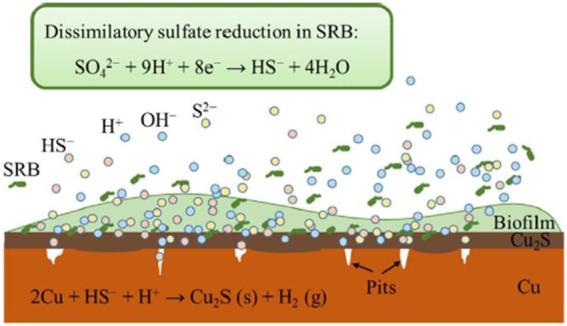
Schematic representation of copper corrosion mediated by sulfate-reducing bacteria (SRB) activity. Obtained from Dou et al. (2020).

As in the case of steel-based materials, sulfide is the main corrosive agent of copper materials. According to Dou et al. (2020), H_2_S produced by SRB cause uniform corrosion accompanied by pitting corrosion of Cu (high concentration of H_2_S) or intergranular corrosion of this metal (low levels of H_2_S). In this study, the dissimilatory reduction of sulfate mediated by SRB produced HS^-^ from the secreted H_2_S. This metabolite can be decomposed to H^+^ and S^2-^ or when combined with H^+^ forms H_2_S. The HS^-^ diffuse to Cu surface and reacts with it resulting in Cu_2_S (Figure 3). Chen et al. (2011) described the different phases of the anaerobic corrosion of Cu starting with the formation of a chemisorbed Cu^I^ surface state acting as a precursor to forming a film. In the presence of SH^-^ in the medium, it can bind to Cu producing the chemisorbed species of Cu (SH)_ads_. SH^-^ can then react with the Cu surface and the adsorbed species leads to the beginning of the formation of a Cu_2_S film with partial protective capacity. In presence of Cl^-^, it can react with the surface intermediate Cu (SH)_ads_ forming a chloride complex (CuCl_2_^-^). This compound can lead to Cu^+^ transport through the pores to the Cu_2_S/solution interface and react with SH^-^ to form Cu_2_S. Several studies have shown the presence of chalcocite (Cu_2_S; Pedersen, 2010; Chen et al., 2014; Kurmakova et al., 2019) and surface pits (Chen et al., 2014) on the surface of oxygen-free copper under anaerobic conditions mediated by SRB.

As we have seen, the MIC rate of each material mainly depends on the presence of the sulfide produced by the activity of anaerobic microorganisms such as SRB. In a DGR, the corrosion of metal canisters will probably depend on the sulfide concentration at the boundary between the groundwater and the compacted bentonite (Bengtsson and Pedersen, 2016). Moreover, MIC may not only affect the release of radionuclides from canister, but could also be involved in gas production within the DGR system, which will be discussed in the next section.

### 4.2. Gas formation by microorganisms

As mentioned before, a wide variety of organic and inorganic compounds present in the DGR environment will provide nutrients, electron donors and acceptors, carbon, nitrogen, and sulfur sources which would enhance microbial activity. Not only some physicochemical processes such as corrosion of metal canisters or the radiolysis of water, but also microbial metabolism may contribute to the build-up of a gas phase in DGR environments (Guo and Fall, 2021). Gas accumulation derived from microorganisms is mainly produced in form of hydrogen (H_2_), methane (CH_4_), and carbon dioxide (CO_2_), and can lead to an increased pressure within the repositories, compromising the integrity of the clay as barriers (Bagnoud et al., 2016b).

H_2_ generation is one of the main gaseous sources within DGRs and may be conducted by the anoxic corrosion of the metal canisters, radiolysis of water, and diverse microbial activities (Beaton et al., 2019; Hall et al., 2021). Although the production of this gaseous form can increase the pressure level, some microorganisms can use it as a source of energy for microbial growth by means of methanogenesis or sulfate reduction (Grigoryan et al., 2021; Hall et al., 2021). CH_4_ can be produced through microbial methanogenesis through reactions between H_2_ or H_2_O and inorganic carbons (CO_2_ and CO) or acetate fermentation (CH_3_COOH; Eqs. 1, 2; Chen et al., 2019; Abramova et al., 2023). Methanogenesis is assumed to decrease gas pressure in DGR surroundings by consuming both CO_2_ and H_2_ (Eq. 1; Leupin et al., 2017; Beaton et al., 2019). However, the production of both CH_4_ and CO_2_ derived from aceticlastic methanogenesis (Eq. 2) could play the opposite role.

(1) $$4\;H_{2}+{\mathrm{CO}}_{2}\to {\mathrm{CH}}_{4}+2\;H_{2}O$$

(2) $${\mathrm{CH}}_{3}\mathrm{COOH}\to {\mathrm{CH}}_{4}+{\mathrm{CO}}_{2}$$

Recently, metagenomic and metaproteomic analyses on the microbial community present in Opalinus clay under DGR simulated conditions, have been performed to determine the role of microbial metabolic pathways on the safety of DGRs. These analyses indicate the high contribution of the bacterial genus *Pseudomonas*, as an SRB, in the oxidation of H_2_ coupled with sulfate (SO_4_^2-^) reduction (Eqs. 3, 4; Bagnoud et al., 2016a; Smart et al., 2017). SRB from the family *Peptococcaceae* also appear to be involved in the formation of H_2_S as a result of the respiration of sulfates and CO_2_ from the oxidation of acetates and other organic compounds (Eq. 5; Bagnoud et al., 2016a). Finally, the CO_2_ produced as a result of some of the biochemical reactions mentioned above can dissolve in pore water, precipitated through its interaction with cementitious materials or diffused in a gaseous form, depending on the area where it is produced within the storage facilities (Bagnoud et al., 2016a).

(3) $${\mathrm{SO}}_{4}{}^{2-}+4\;H_{2}\to H_{2}S+2\;{\mathrm{OH}}^{-}$$

(4) $${\mathrm{SO}}_{4}{}^{2-}+4\;H_{2}\to S^{2-}+2\;H_{2}O$$

(5) $${\mathrm{SO}}_{4}{}^{2-}+{\mathrm{CH}}_{3}\mathrm{COOH}\to H_{2}S+2\;{\mathrm{HCO}}^{3-}$$

As commented in previous sections, the presence of methanogenic and sulfate-reducing microbes has been detected in several clay types that will be used in DGR systems. For this reason, it is crucial to investigate in depth the influence of their metabolism on the safety of deep disposal. As different gases are consumed and produced during all these mechanisms, it is important to quantify the *in-situ* rates of microbial H_2_ oxidation and SO_4_^2-^ reduction to determine the total net production of gases. The calculations of Bagnoud et al. (2016c) concluded that sulfate-reducing microorganisms from Opalinus clay could be beneficial for the safety of the geological disposal of nuclear waste as indicated by high hydrogen and sulfate consumption rates, which would reduce the gas pressure build-up. However, it is not easy to estimate the overall role of microbes in terms of security since very different chemical, biological, and physical processes may be involved. For instance, the removal of sulfate gases by SRB may induce steel corrosion of the containers through the production of H_2_S or sulfides (S^2-^) as we discussed in section 4.1.

To sum up, microorganisms could both positively and negatively influence the safety of DGRs as far as the utilization and production of gases is concerned. However, it is extremely difficult to determine their overall impact due to the complexity of the biogeochemical processes that will be involved. In addition, the final role of microorganisms in terms of gas production will depend partly on the microbial community structure present in the natural and artificial barriers surrounding the canisters. Further research on microbial metabolism and gas formation is crucial to evaluate the safety conditions of DGRs.

### 4.3. Bentonite biogeochemical transformations

One of the materials selected as an artificial filling and sealing barrier by current DGR models is bentonite, a volcanic origin phyllosilicate. This type of clay is mainly composed of smectites, highlighting montmorillonite as the principal mineral phase. Montmorillonite is characterized by a layered structure consisting of each one of 3 sheets in a 2:1 ratio, i.e., two tetrahedral silica sheets bordered in between them by an octahedral aluminum sheet (T-O-T structure). These layers have a negative charge balanced by cation exchange, providing the bentonite with most of its physico-chemical properties (Abdullahi and Audu, 2017; García-Romero et al., 2019). Bentonite from multiple locations has been widely studied as a buffer material for DGRs (MX80 from United States, FEBEX from Spain, FoCa from France and GMZ from China, among others; Xu et al., 2019).

Both abiotic (temperature, radiation, etc.) and biotic factors could compromise the stability of the bentonite barrier and therefore jeopardize the safety of the system. During the first phases of the repository, high temperatures are expected to be reached due to the heat generated by the decay of the stored radionuclides (Tripathy et al., 2017). The heat would diffuse throughout the repository reaching up to 100°C–200°C in the bentonite buffer with a maximum temperature gradient of up to 24°C, over a thickness of about 35 cm (Hökmark and Fälth, 2003; Faybishenko et al., 2017; Zheng et al., 2017). Smits et al. (2013) supported that an increase in temperature means an increase in thermal conductivity, probably due to an additional heat transfer as latent heat. However, this temperature effect decreases with high bentonite dry density. High temperatures are also a matter of great concern as they could lead to an abiotic smectite illitization process. Mills et al. (2022) discussed a possible mechanism of illitization at 200°C that would occur through a layer-by-layer transformation. The individual phyllosilicate layers are enriched in charge due to the substitution of Si by Al in the tetrahedral sheet, the substitution of Al by Mg in the octahedral sheet and an interlayer cation exchange of K^+^ by Na^+^ resulting in the formation of mixed illite/smectite layers. This phenomenon results in chemical alterations which could affect bentonite properties such as swelling and plasticity capacities (Zheng et al., 2017). In addition to temperature, bentonite will inevitably be exposed to certain doses of ionizing radiation, mainly gamma (from Cs-137). Many studies have focused on characterizing the effect of γ-radiation on this clay and reported only negligible effects on the alterations of its physical and chemical properties (Pushkareva et al., 2002; Plötze et al., 2003; Huang and Chen, 2004). However, Holmboe et al. (2011) tested the effect of this radiation on the ability of MX80 bentonite to retain radionuclides such as Cs^+^ and Co^2+^. Only Co^2+^ sorption was significantly affected by γ-radiation, which decreased in the irradiated samples. This result could indicate that this kind of irradiation would have altered surface characteristics thus decreasing its ability to bind this radionuclide. However, some authors have reported the stability of bentonite mineralogy when treated with different solutions (e.g., distilled water, sodium nitrate, glycerol-2-phosphate, and uranyl nitrate) after 6 months of aerobic incubation (Povedano-Priego et al., 2019). XRD semi-quantitative estimation showed the same composition of smectite, quartz, phyllosilicates, and plagioclases. Smectite represented the dominant mineral phase with 91%. Similar results were obtained for bentonite microcosms incubated for 6 months but under anoxic conditions, which indicates the stability of bentonite and no illitization process under different short-term incubation (Povedano-Priego et al., 2022).

However, the present review focus mainly on biotic factors related to the presence of microorganisms in the different barriers. One of the main concerns related to bentonite buffer is the ability of microorganisms to interact with the minerals causing weathering, dissolution and a second mineral formation (Meleshyn, 2014). In particular, smectite biotransformation into illite through Fe (III) bio-reduction is one of the most alarming processes. Pedersen (2013) reported that both the hydrogen sulfide produced by SRBs (Masurat et al., 2010), and Fe (II) generated by iron-reducing bacteria (IRB) could lead to a possible illitization process that would alter the properties of the smectite. Furthermore, Kim et al., 2019 studied the ability of the strain *Shewanella oneidensis* MR-1 to induce smectite dissolution by Fe (III) bio-reduction processes. Numerous studies reviewed by Dong et al. (2009), demonstrated the bioreduction of Fe (III) by microorganisms in clay minerals. The iron bioreduction rate was related to different factors, including total Fe content in the bentonite, particle size, amount of microorganisms present, pH, etc. (Lopez-Fernandez et al., 2021).

### 4.4. The influence of microorganisms in radionuclide mobilization/immobilization

Radionuclides cannot be entirely removed, but they can be transformed to lower toxicity forms. Microorganisms are known for their capacity to do so, largely due to their high tolerance to toxic elements and other stressful conditions including radiation, desiccation, and the presence of oxidative agents (Brim et al., 2000). A wide variety of bacteria have been previously described to efficiently resist toxic elements such as uranium, nickel, copper, cadmium, selenium, cesium, strontium, etc., including those belonging to the genera *Bacillus, Pseudomonas,* or *Stenotrophomonas* (Fakhar et al., 2022; Ruiz-Fresneda et al., 2023).

**Figure 4:**
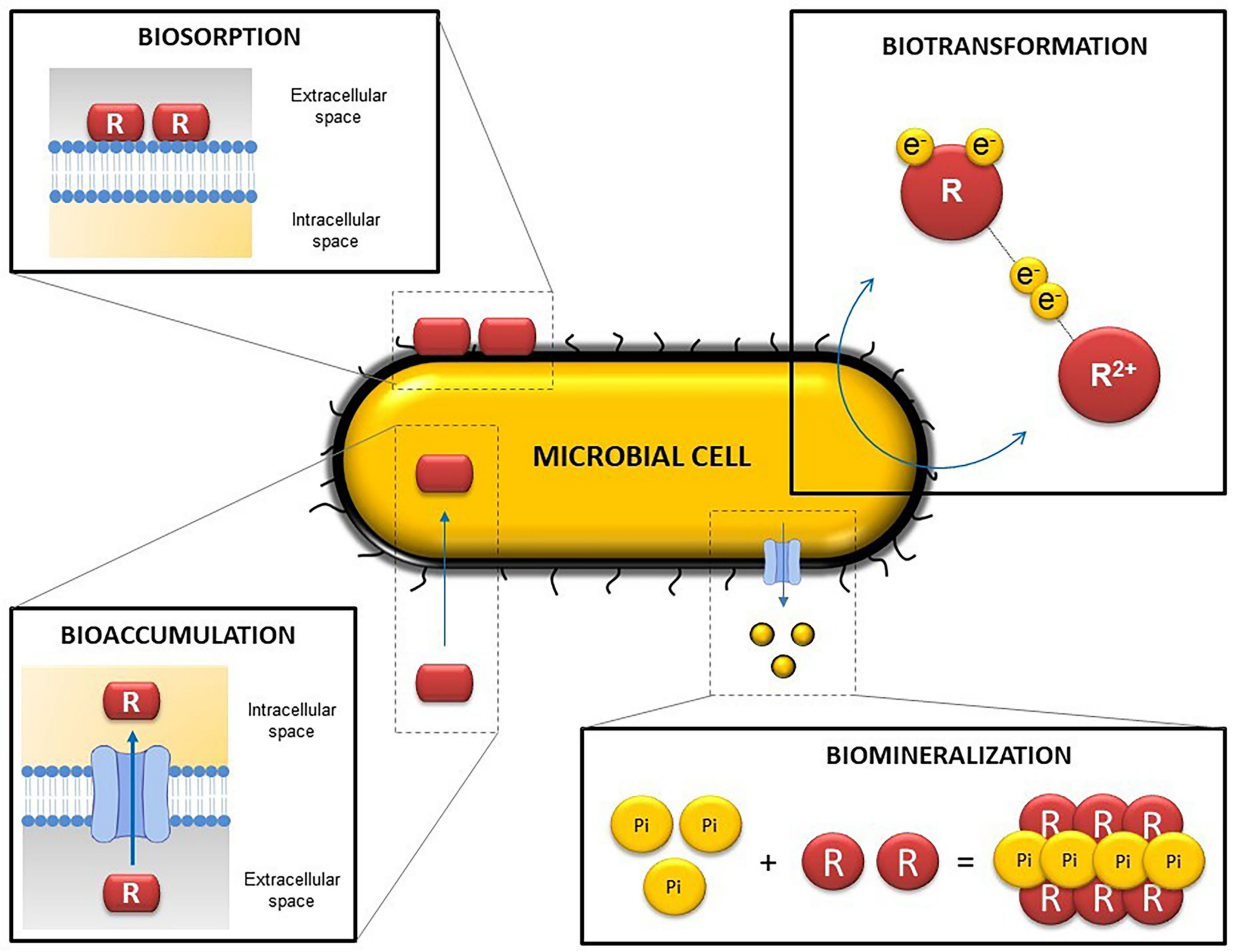
Representative diagram illustrating the main microbial mechanisms involved in radionuclide and heavy metal mobilization. R, radionuclide/ligand.

Microbial activity can indirectly influence solubility and hence the mobilization of radionuclides by the alteration of the geochemical conditions within the DGRs (pH, oxidation–reduction potential, etc.). Such modifications can lead to changes in the oxidation state of some radionuclides, affecting their solubility and mobility through the repositories. However, microorganisms can directly influence the mobility of radionuclides and other elements through different processes such as intracellular accumulation, biotransformation (redox reactions) biosorption, or biomineralization (Shukla et al., 2017; Lopez-Fernandez et al., 2020; Ruiz-Fresneda et al., 2020a; Martinez-Rodriguez et al., 2022; Figure 4).

Biotransformation occurs when a specific element changes its oxidation state as a result of microbial activity through redox reactions and can lead to changes in its solubility (Francis and Nancharaiah, 2015). The bioreduction of soluble and mobile oxidized forms can trigger their immobilization (Rui et al., 2013). Enzymatic reduction of radionuclides and other toxic elements by bacteria have been widely reported in recent years. Several bacterial species are able to anaerobically reduce the soluble form U^VI^, to the insoluble U^IV^, by using U^VI^ as the final electron acceptor (Kolhe et al., 2018). Bacterial genera such as *Geobacter, Desulfovibrio, y Shewanella* are remarkable bacterial genera in uranium reduction (Cologgi et al., 2014; Stylo et al., 2015; Grouzdev et al., 2018). The bioreduction of oxidized forms of other elements have also been described. Se^VI^ and Se^IV^ can be reduced to less mobile forms (Se^0^ and Se^-II^), among others, by the genera *Bacillus* and *Stenotrophomonas* (Tan et al., 2016; Lampis et al., 2017; Kora, 2018; Ruiz-Fresneda et al., 2020a). Volatilization is another biotransformation process considered as a rising green process because of it generally implies the production of non-toxic compounds through reduction and methylation reactions.

Biosorption can be defined as the binding of positively charged metal ions to negatively charged cell surface components through physico-chemical interactions (Gadd, 2004; Prakash et al., 2013). Specifically, it can occur by electrostatic interactions, ionic exchange, or complexation and chelation processes (Yang et al., 2015; Diep et al., 2018). For this reason, biosorption is considered a passive process and can even take place with cellular fragments (cell membranes, cell walls, etc.) and dead microbial biomass (Fomina and Gadd, 2014; Ayangbenro and Babalola, 2017). Biosorption can act directly between metal cations and anionic functional groups from cell walls, or indirectly with extracellular polymeric substances (EPS), S-layer proteins, and the bacterial capsule (Merroun et al., 2005; Dobrowolski et al., 2017).

According to Tabak et al. (2005), bioaccumulation consists of accumulating a specific substance or element inside an organism. In contrast to biosorption, intracellular accumulation is usually considered a metabolically active process, in which microorganisms introduce radionuclides or other elements into the intracellular space by using a transport system or altering the permeability of the membrane (Diep et al., 2018). However, some authors indicate that bioaccumulation is closely related to biosorption, since it requires fast interactions with anions from components of the cell surface before being introduced into the intracellular space (Tišáková et al., 2013). For this reason, other authors have considered that intracellular accumulation can occur through both passive sorption (metabolism-independent) and active uptake (metabolism-dependent). Once the radionuclide is inside, it can be sequestered by proteins, packaged in lipid membranes, vacuoles, or different biological storage systems (Mishra and Malik, 2013).

Bioprecipitation is based on the precipitation of elements with ligands (phosphates, sulfides, carbonates, hydroxides, etc.) released by microorganisms resulting in the production of insoluble and immobile complexes (Shukla et al., 2017; Sreedevi et al., 2022). Some bacterial species from the genera *Sphingomonas, Bacillus* and *Stenotrophomonas* have the capacity to release inorganic phosphates (Pi) through phosphatase activity, which precipitates with U^VI^ and produce insoluble uranium phosphates (Chandwadkar et al., 2018; Zhang et al., 2018; Sánchez-Castro et al., 2020; Zhong et al., 2021). When the complex formed is a mineral this bioprecipitation process is called biomineralization (Kolhe et al., 2018). It is important to note that the metals or radionuclides precipitated as a result of this process do not present any change in their oxidation states, unlike other processes formerly described, such as biotransformation.

Tolerance of microbes to radiation must be considered of particular interest in microorganism-radionuclide interaction. It is well known that radiation can both directly and indirectly affect bacterial biomolecules, including nucleic acids, proteins, lipids, and carbohydrates. For instance, ionizing particles produced as a consequence of radioactivity can damage the DNA structure, disrupting the functionality of the DNA (Close et al., 2013; Jung et al., 2017). Ionizing radiation can also indirectly damage the microbial DNA and other molecules by production of reactive oxygen species through the radiolysis of water, as discussed in Section 2. To neutralize radiotoxicity, microorganisms have developed defense mechanisms, such as the production of regulatory secondary metabolites, DNA repairing machineries, anti-oxidative systems, etc. (Gabani and Singh, 2013). This is the case of ionizing radiation-resistant bacteria such as *Deinococcus radiodurans*, which tolerate high radiation doses (12 kGy), *Kineococcus radiotolerans* (2 kGy), or *Rubrobacter radiotolerans* (1 kGy), among others (Jung et al., 2017). However, microorganisms do not implicitly need to tolerate high radiation doses to play a positive role in DGR safety. As mentioned before, radionuclide biosorption is an electro-chemical passive process, in which the cells do not have to be viable to successfully interact with the radionuclides. Even when the viability is almost non-existent in a bacterial population, their metabolism could remain active under stress situations. Indeed, some studies have revealed that less than 2% of the cells of the bentonite isolate *S. bentonitica* are viable under DGR simulated conditions (anaerobiosis, Se^IV^ stress, etc.) after 6 days’ incubation, but more than 50% remain metabolically active and interact with Se^IV^ through its biotransformation to Se^0^ (Ruiz-Fresneda et al., 2019). In addition, radiation will be strongly attenuated and absorbed in the proximity of the DGRs overtime and the help of insulating materials and multi-barrier systems. Nevertheless, when we do not specifically know when the interaction may occur (whether the radiation is active or not), we must study as many conditions as possible in all the interaction cases.

## 5. Interaction mechanisms between microorganisms and relevant radionuclides

Despite heavy metal-bacteria interaction having been studied in depth during the last decades, not many studies have been reported in relation to the possible role of these interactions influencing the safety of DGRs in the case of a radionuclide escape. A lot of effort has been put into very interesting studies which have been published in recent years with the aim of elucidating as far as possible how microbial processes can affect the mobility of elements of interest present in radioactive waste. These studies will allow us to evaluate how microorganisms can positively or negatively affect the safety of future DGRs. In this review, we have focused on Se, as one of the critical radionuclides of radioactive waste, and some representatives of trivalent actinides, such as Cm and their inactive analogs (trivalent lanthanides such as Eu).

### 5.1. Microbial interactions with selenium

**Figure 5:**
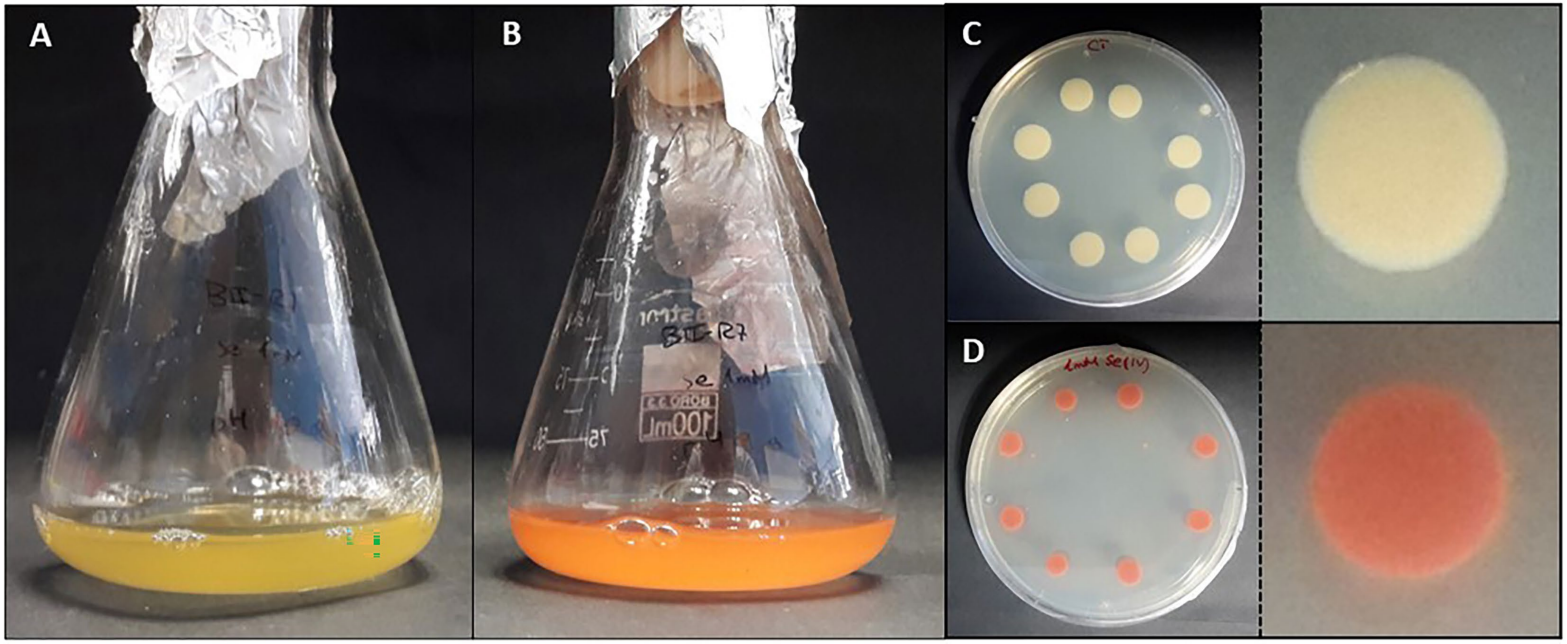
Colorful transition of liquid **(A,B)** and solid **(C,D)** cultures of the bacterium *Stenotrophomonas bentonitica* to red Se^**0**^ when Se^IV^ is added (personal archive of Dr. Merroun).

Microorganisms can interact with Se mainly through biochemical biotransformation processes. There are many which are capable of transforming different species of Se by means of reduction–oxidation (redox) reactions, methylation, and demethylation processes (Eswayah et al., 2016). The microbial reduction of oxidized and soluble forms of Se (Se^VI^ and Se^IV^) to insoluble Se^0^ has been previously described by bacteria of the genera *Bacillus, Shewanella, Comamonas, Stenotrophomonas, Azospirillum,* and *Pseudomonas*, among others (Figure 5; Kora, 2018; Vogel et al., 2018; Baggio et al., 2021; Staicu et al., 2022; Ruiz-Fresneda et al., 2023). Currently, the number of known Se^VI^ reducing strains is lower than that of Se^IV^ reducing ones. The mechanism of reduction of Se^VI^ varies among the microorganisms studied to date. Some bacterial species such as *Thauera selenatis* and *Seleniivibrio woodruffii* are able to respire Se^VI^ when using it as an electron acceptor (Rauschenbach et al., 2013; Mohapatra et al., 2022). On the other hand, some enzymes such as selenate reductase have shown their ability to reduce Se^VI^ in strains such as *Comamonas testosteroni* S44 (Tan et al., 2018). The reduction of Se^IV^ can also be carried out through different enzymatic mechanisms. Nitrite reductase, sulfite reductase, fumarate reductase and selenite reductase seem to be involved in this process (Song et al., 2017; Wang et al., 2018, 2022). Furthermore, compound-mediated reactions with thiol groups (-SH) have also been studied. The participation of glutathione (GSH) in the reduction of Se^IV^ has been investigated with special interest. Kessi and Hanselmann (2004) found that GSH and the enzyme glutathione reductase (GR) are involved in the reduction of Se^IV^ to Se^0^ in *Rhodospirillum rubrum* through a series of reactions. Recently, the studies of Wang et al. (2022) conducted with the strain *Proteus penneri* LAB-1 support this mechanism by suggesting that the gluthathione pathway plays a critical role in the Se^IV^ reduction process carried out by this bacterium.

In most cases, the reduced Se^0^ products are accumulated in the form of selenium nanoparticles (SeNPs), known for their use in numerous medical and industrial applications (Zambonino et al., 2021). These nanoparticles are characterized by different physico-chemical properties (morphology, size, structure, etc.), that could affect their solubility and mobility in the environment (Jain et al., 2017). For example, *Bacillus subtiliis* BSN313, *Bacillus mycoides* SeITE01, and *Stenotrophomonas maltophilia* SeITE02 are able to produce Se spheres with an amorphous nature at the nanoscale (Bulgarini et al., 2021; Ullah et al., 2021). The studies by Benko et al. (2012) demonstrated the lower toxicity of Se nanospheres compared to the oxidized forms (Se^VI^ and Se^IV^). However, there is some controversy in this respect since different studies have indicated completely the opposite (Li et al., 2008). The formation of Se nanowires by microorganisms present in anaerobic granular sludge has also been proved by Jain et al. (2017). These authors have described the lower colloidal stability and mobility of these nanostructures compared to biologically produced nanospheres. On the other hand, the crystallinity of some Se nanostructures also seems to affect their immobilization by increasing their settleability (Lenz et al., 2009).

**Figure 6:**
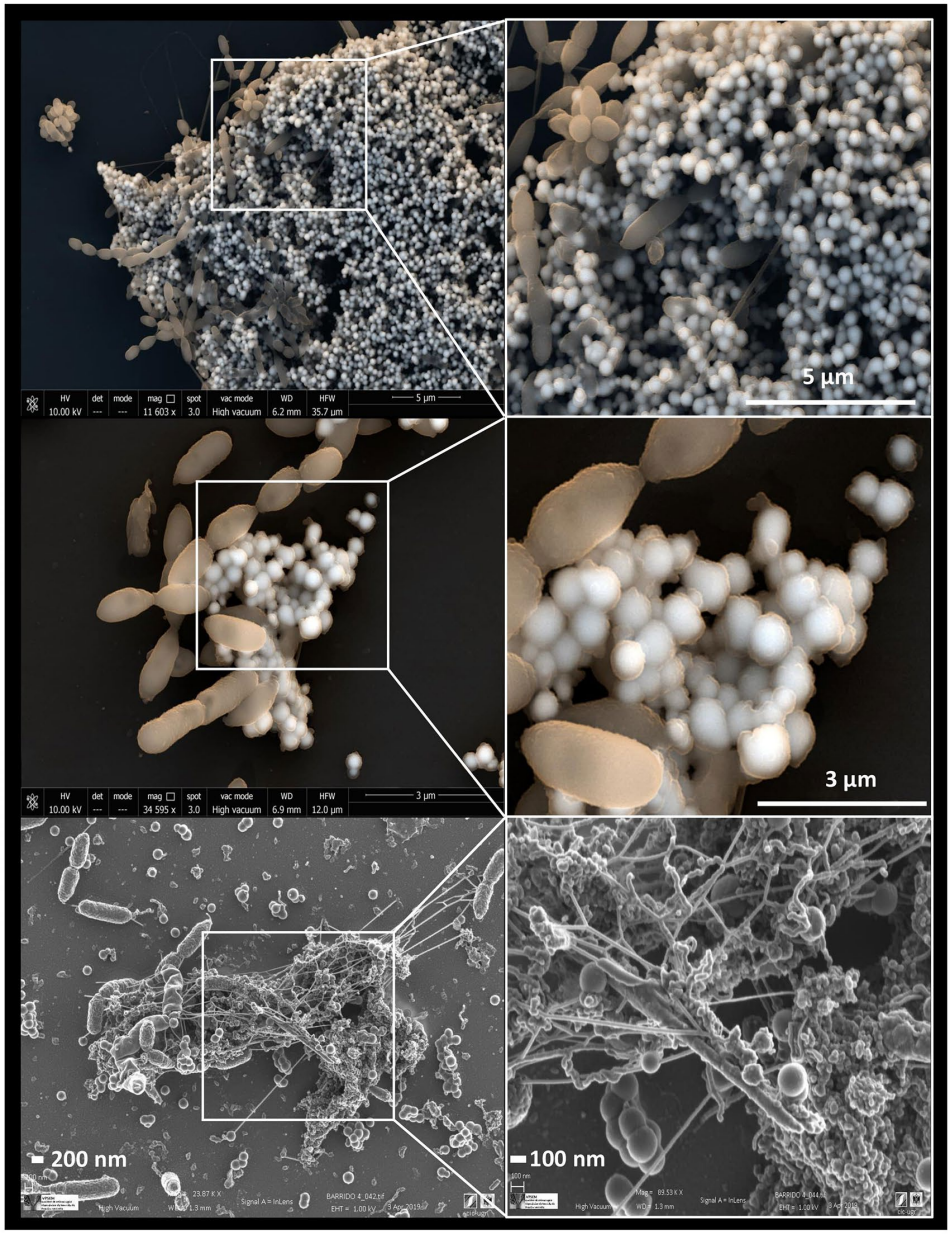
Electron microscopy images showing Se^0^ nanostructures produced by the bacterium *Stenotrophomonas bentonitica* when supplemented with Se^IV^ (personal archive of Dr. Merroun; growth conditions detailed in Ruiz Fresneda et al., 2018, 2019, 2020a).

Some studies have revealed the presence of organic matter layers, composed mainly of proteins and polysaccharides surrounding the biologically produced Se nanostructures (Dobias et al., 2011; Kamnev et al., 2017; Bulgarini et al., 2021). The physico-chemical properties, and therefore the mobility of Se nanostructures can be largely affected by the presence of these associated organic layers (Jain et al., 2017). In addition, some authors have also suggested the role of proteins in the synthesis and transformation of SeNPs, as well as in controlling their size (Dobias et al., 2011). According to Ruiz-Fresneda et al. (2020a), proteins produced by the bacterium *S. bentonitica* maybe involved in the transformation of amorphous nanospheres to crystalline trigonal Se nanofibers (Figure 6). Although the specific transformation mechanism is still unknown, what seems clear is the direct role of the cells and their proteins during the process.

In the same way as for Se^0^, methylated selenide compounds are basically insoluble and poorly bioavailable for living beings (Favorito et al., 2021). For this reason, Se methylation is considered one of the most important transformation processes related to bioremediation. It is a detoxification mechanism for microorganisms since volatile Se methylated compounds are notably less toxic than the oxidized forms (Davis et al., 2013; Favorito et al., 2021). The production of methylated selenides has been proved in different bacterial species such as *Methylococcus capsulatus*, *Methylosinus trichosporium* OB3b, or *Pseudomonas tolaasii* (Eswayah et al., 2017; Liu et al., 2021). These bacterial strains can produce volatile compounds such as dimethyl selenide (DMSe, CH_3_SeCH_3_), dimethyl diselenide (DMDSe, CH_3_SeSeCH_3_), and dimethyl selenenyl sulfide (DMSeS, CH_3_SeSCH_3_). To date, several mechanisms have been proposed for the biomethylation of Se. Chasteen and Bentley (2003) suggested that Se^0^ is reduced to the selenide form (H-Se-X), which is subsequently methylated to CH_3_SeCH_3_ and CH_3_SeH. Other mechanisms similarly propose the formation of CH_3_SeCH_3_ from Se^IV^ through numerous reduction and methylation reactions (Challenger, 1945). Finally, not many studies have reported the oxidation of Se^0^ and organic Se by microorganisms present in soils. Very recently, Luo et al., 2022 discovered four bacterial strains (*Dyella* sp. LX-1 and LX-66, and *Rhodanobacter* sp. LX-99 and LX-100) capable of oxidizing Se^-II^ and Se^0^ to Se^IV^. However, unlike reduction processes, the low oxidation rate of Se reduced forms indicates that this process should not be considered as relevant for the environment (Losi and Frankenberger, 1998; Eswayah et al., 2016). Similarly, demethylation processes of Se compounds are not usually considered due to the low rates at which these reactions occur (Eswayah et al., 2016).

On the basis of all the comments mentioned above, it is clear that indigenous microorganisms from natural and engineered barriers in a DGR or are introduced during the disposal construction and could play a direct effect on radionuclide mobilization. For example, the bacterial species *S. bentonitica*, isolated from bentonite clay, has been recently described to efficiently reduce Se^VI^ and Se^IV^ to Se^0^ nanostructures and methylated Se (Pinel-Cabello et al., 2021a; Ruiz-Fresneda et al., 2023). The physico-chemical characterization of these Se reduction products suggests that this bacterium plays an important role in Se immobilization in the context of DGRs. Recently, Povedano-Priego et al. (2023) have described the Se^IV^ reduction to Se^0^, producing precipitates of different shapes (from nanospheres to complex nanostructures) in bentonite samples treated with Se^IV^. This is the first time that this process has been shown within a ternary system (bentonite, indigenous microorganisms, and Se). Several bacteria have been found enriched in Se^IV^-treated bentonite such as *Pseudomonas*, *Stenotrophomonas*, *Desulfosporosinus*, among others, potentially involved in Se reduction (Povedano-Priego et al., 2023).

### 5.2. Microbial interactions with Cm/Eu

Different interaction mechanisms such as biosorption, biomineralization/bioprecipitation and intracellular accumulation have been described as affecting the mobility of representatives of trivalent actinides such as Cm, and their inactive analogs including Eu.

Biosorption is one of the main processes involved in the case of Cm and Eu. The cell surfaces of many bacteria have a high density of functional groups that can serve as a binding site of certain components and metal cations (Hufton et al., 2021). Specifically, the carboxyl groups present in the peptidoglycan layer of the cell wall of gram-positive bacteria seem to act as the main binding sites for actinides (Barkleit et al., 2009). Moreover, carboxyl, phosphoryl, and hydroxyl groups present on lipopolysaccharides (LPS) of the outer membrane, characteristic of gram-negative bacteria, can also act as a ligand for actinides such as U, Cm, and Np (Moll et al., 2021). Different bacterial species such as *Pseudomonas fluorescens* and *Sporomusa* sp. MT-2.99 have been described for the efficient biosorption of Cm^III^ and Eu^III^ to their cell surfaces (Moll et al., 2013, 2014). *Pseudomonas fluorescence* was isolated from groundwater from the Äspö Hard Rock Laboratory tunnel, a unique research facility testing the safety of final repositories for nuclear fuel, while *Sporomusa* sp. MT-2.99 is indigenous from Mont Terri Opalinus clay. In the case of *Sporomusa* sp. MT-2.99, the speciation and structure of the surface complexes formed with Cm^III^ and Eu^III^ was determined by Time Resolved Laser-Induced Fluorescence Spectroscopy (TRLFS). The results show that carboxyl and phosphoryl groups are responsible for Eu^III^ and Cm^III^ binding and the cell surface of this bacterium (Moll et al., 2014). Recent new research has demonstrated the role of proteins from the plasma membrane fractions of *L. sphaericus* in Cm^III^ complexation (Moll et al., 2021).

**Figure 7:**
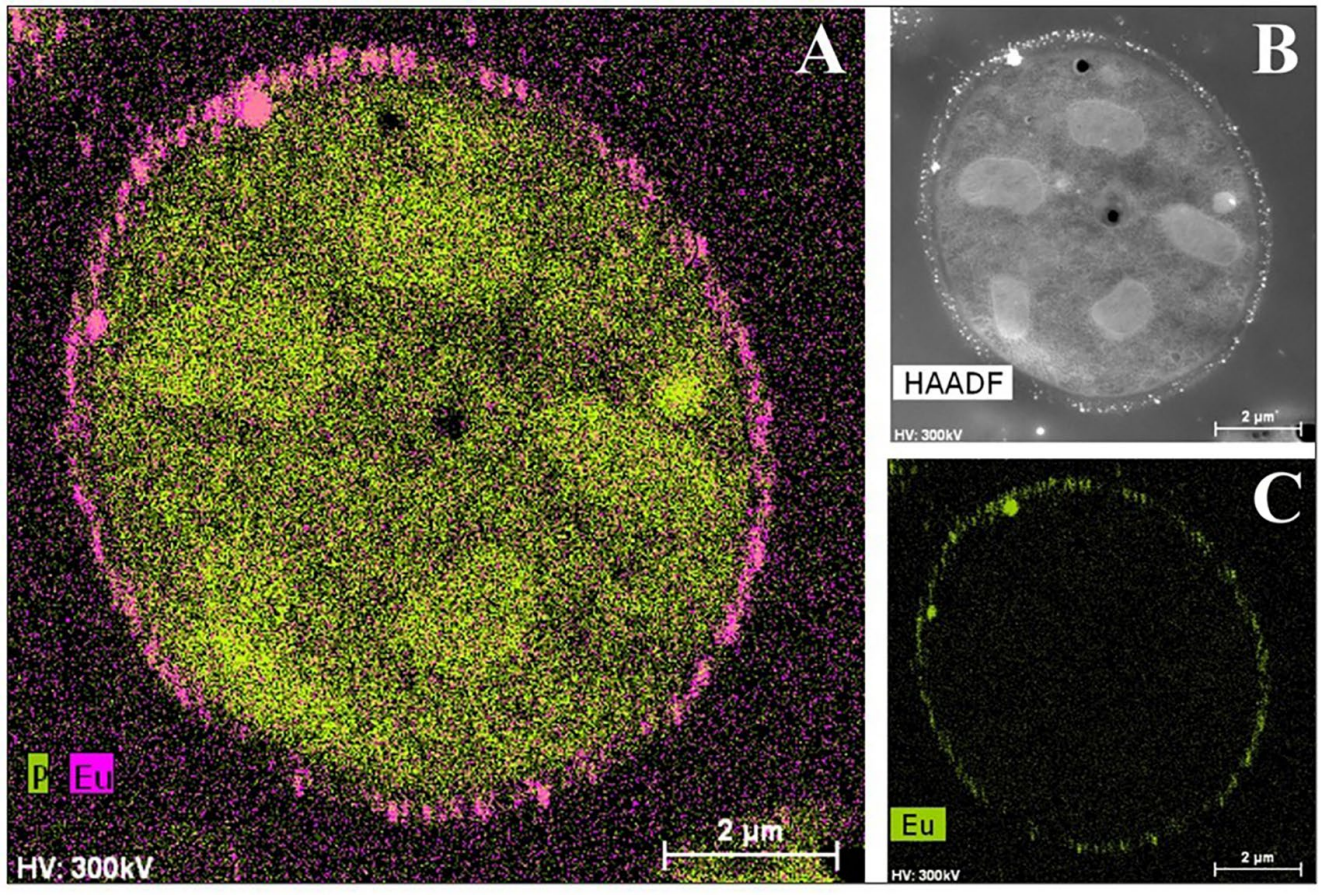
HAADF-STEM micrographs of a thin section and EDX element distribution maps showing the Eu biosorption to the cell surface of *Rhodotorula mucilaginosa* BII-R8. **A**: P and Eu elemental mapping image; **B**: HAADF image; **C**: Eu elemental mapping image (Lopez-Fernandez et al., 2018c).

Not only have bacterial cells been analyzed using this technique, but there have also been recent studies about the yeast *Rhodotorula mucilaginosa* BII-R8, isolated from selected Spanish bentonite, which has shown its ability to retain both Eu^III^ and Cm^III^ at the cell surface Eu^III^ (Lopez-Fernandez et al., 2018c; Figure 7). In the same way, carboxyl and phosphoryl groups present in the cell envelopes of *R. mucilaginosa* BII-R8 seem to be involved in the interaction of these two elements according to the results obtained by TRLFS. This study described for the first time the ability of a yeast to efficiently interact with Cm^III^ and Eu^III^. Archaea can also interact with Cm^III^ and Eu^III^ as indicated by studies on *Halobacterium noricense* DSM15987T (Bader et al., 2019), where phosphate and carboxylic groups from the cell surface and released by the cells most probably act as binding sites. *H. noricense* DSM15987T was isolated from rock salt, one of the natural barriers being considered for radioactive waste disposal.

Bioaccumulation and biomineralization/bioprecipitation have also played an important role in the microbial interaction with Cm^III^/Eu^III^. The bacterial species *Thermus scotoductus* SA-01 accumulates Eu^III^ precipitates both intracellularly and extracellularly and tolerate higher concentrations than those usually found in nature (up to 1 mM; Maleke et al., 2019). Analysis of these samples by various spectroscopic techniques suggest the presence of extracellular accumulation may be due to a biomineralization process from carbonates produced by the reaction of CO_2_, from respiration, with OH^–^ radicals. They have also demonstrated the possible role of carboxyl, carbonyl, and phosphate groups in the biosorption of Eu^III^ according to the data obtained by ATR-FTIR. Specifically, they suggest the formation of Eu^III^ carbonates at surface level as indicated by XPS analyses. These results show how several mechanisms of interaction can simultaneously take place in a cell when it is exposed to a stressful condition. This is also the case of *S. bentonitica*, a bentonite-isolated bacterium which has recently been reported to interact with Eu^III^ and Cm^III^ through biosorption and bioaccumulation mechanisms, and possibly biomineralization (Ruiz-Fresneda et al., 2020b). The ability of this bacterium to additionally interact with Se, as mentioned before, suggests it as a potential positive candidate for the immobilization of radionuclides in the context of DGRs.

To sum up, microbial biosorption and bioaccumulation processes may play an important role in the immobilization of Cm^III^, Eu^III^, and other toxic elements if microbial biomass binds to inert supports through the formation of biofilms (Gadd, 2009). Biomineralization may also lead to the immobilization of these elements due to the formation of insoluble precipitates (Shukla et al., 2017). For all these reasons, the study of microbial interactions with actinides (Cm^III^) and analogous elements (Eu^III^) has been acquiring a special interest in recent years, both for the evaluation of the safety of radioactive waste repositories, and for the bioremediation of contaminated environments.

### 5.3. Microbial interaction with U

Uranium will be the dominant and the most critical radionuclide in the DGR system. Interactions between bentonite microbial communities and such radionuclide could be an imperative solution for the retention and/or immobilization, avoiding a release of U^VI^ in the environment and allow a safe and long-term storage of the radioactive waste.

The mobility and solubility of U in natural systems are governed by its oxidation state as well as its chemical speciation, which in turn is influenced by abiotic and biotic processes. U has two major oxidation states, U^VI^ and U^IV^. The oxidized form possesses an elevated number of positive charges that results in its solubility, mobility, and therefore toxicity to the microbial cells, while U^IV^ is insoluble and less toxic under anoxic conditions. The chemical speciation of U^VI^ is highly pH-dependent, in the sense that under alkaline conditions, such as those of bentonite, soluble uranyl ions it may form complexes with carbonate groups (Waite et al., 1994; Kushwaha et al., 2018), which are potentially mobile in presence of water. Under acidic conditions these oxidized forms would be absorbed on the mineral phases, complexed by organic matter or precipitated as autunite [Ca(UO_2_)_2_(PO_4_)_2_], one of the insoluble uranyl phosphate minerals (Kolhe et al., 2018). In addition to chemical toxicity, U also displays a radiological (mainly from ^235^U and ^238^U isotopes) hazard, although its radiotoxicity is reported to be low. In contrast to other metals, it does not have any biological functions in living organisms. However, it has long been known to cause lung, renal, and hepatic damage in humans (Priest, 2001), as it may provoke oxidative stress in lung epithelial cells, by the loss of glutathione (an effective antioxidant) and superoxide dismutase enzyme (Periyakaruppan et al., 2007). In microorganisms, many effects of U toxicity are recognized including the loss of cell viability and activity, distortion of cell surfaces, oxidative damage, as well as the suspension of DNA replication and transcriptional and translational processes (Park and Jiao, 2014; Sepulveda-Medina et al., 2015). However, as mentioned above, in order to resist metal and radionuclide toxicity, microbes have developed a variety of mechanisms that allow potential immobilization and bioremediation. Microorganisms that interact with U are reported to be a good remediation strategy for decreasing the solubility and mobility of this toxic element (Sánchez-Castro et al., 2020). They can affect its speciation and migration in different environments, including DGRs (Povedano-Priego et al., 2019, 2022). Among the mechanisms adopted by many microorganisms to tolerate and survive in uranium contaminated sites, biosorption, bioaccumulation, biomineralization, and biotransformation (through oxidation–reduction reactions) constitute the most relevant in the *in situ* and *ex situ* bioremediation techniques. However, the two microbial processes that have been most investigated so far, and therefore gained more attention and confidence, are the enzymatically catalyzed reduction of U^VI^ to U^IV^ and the bioprecipitation of the oxidized form of U with inorganic phosphates as ligands.

Uranium (as oxidized form U^VI^) has shown to be highly susceptible to enzymatic reduction by microorganisms, which can occur in the cytoplasm, periplasm, at the outer membrane or extracellularly (You et al., 2021). They could be involved in a direct enzymatic reduction of soluble U^VI^ to insoluble forms, while biologically mediated indirect reduction using electrons obtained in the oxidation–reduction reactions of iron or sulfate has also been reported to be important in the immobilization of U^VI^ (Roh et al., 2015). Uranium-reducing microorganisms are ubiquitous in the environment. Several species of prokaryotes have been reported to be involved in uranium reduction. Iron-reducing species like *Geobacter uraniireducens*, *G. daltonii, and Shewanella oneidensis* and sulfate-reducing bacteria (SRB) mainly *Desulfovibrio, Desulfotomaculum,* and *Desulfobacterium* have been described as using uranium as an electron donor in addition to iron and sulfate (Akob et al., 2012). The first ones can conserve energy for their anaerobic growth through the reduction of uranium, while the SRB are unable to conserve such energy for their growth. *Desulfovibrio* has been found in different uranium-contaminated sites such as sub-surface sediments (Castañeda-Carrión et al., 2010; Newsome et al., 2014), and water (Parihar et al., 2013; Jroundi et al., 2020). Povedano-Priego et al. (2022) identified *Desulfovibrio* in the bacterial community of uranium-treated bentonite under anoxic conditions in presence of glycerol-2-phosphate (G2P). Significant presence of *Desulfovibrio* in these conditions indicates that a potential U-tolerance mechanism maybe active with the mediation of G2P. The capacity of members of this genus to use glycerol as electron donor has been reported (Ben Ali Gam et al., 2018). In addition, *Desulfovibrio* has been shown to use cytochrome *c3* (*cysA*) functioning as U^VI^ reductase in combination with a hydrogenase as a physiological electron donor, although additional pathways from organic electron donors (such as glycerol) to U^VI^ that can bypass the cytochrome, have been suggested to occur (Payne et al., 2002). Several iron reducers (e.g., *Clostridium*, *Geobacter*) can reduce uranium by different mechanisms mediated by enzymes and cytochromes. Clones related with *Clostridium* species have been detected in the bacterial community of uranium-contaminated sediment from an inactive uranium mine (Midnite mine, eastern Washington), producing the reduction of U^VI^ (Suzuki et al., 2003). The main mechanism involved is an enzymatic reduction of U^VI^ mediated by hydrogenases (Gao and Francis, 2013). Other cytochromes have been found to be imperative to induce uranium reduction in *Geobacter* as well as in other bacteria such as *S. oneidensis*. In addition to the periplasmic c7-type cytochrome PpcA, an important intermediate electron carrier (in the absence of hydrogen), GscA (*Geobacter* sub-surface c-type cytochrome A), MacA (diheme c-type cytochrome peroxidase), MtrC (also known as OmcB; outer membrane c-type cytochrome) and OmcZ (outer-surface c-type cytochrome) has shown to be essential for the reduction of uranium (Yun et al., 2016; Rogiers et al., 2022). Bacterial pili also seem to play a role as an electron conductor between the cells and the electron acceptors. The reduced U^IV^ appears mainly localized in the periplasm and outside of the cells, indicating the involvement of outer membrane-bound enzymes in the reduction process (Majumder and Wall, 2017). It has also been suggested that uranium reduction maybe linked to iron metabolism, since siderophores have been shown to form stable complexes with several metals and radionuclides, including uranium (Gallois et al., 2022). Siderophores and a large number of proteins associated to iron uptake systems, such as ABC-transport type proteins in the Chernobyl isolate *Microbacterium oleivorans* A9 or a transcriptional regulator of the Fur family in *Desulfotomaculum reducens* MI-1, are upregulated as an iron starvation response to uranium stress (Junier et al., 2011; Gallois et al., 2022).

**Figure 8:**
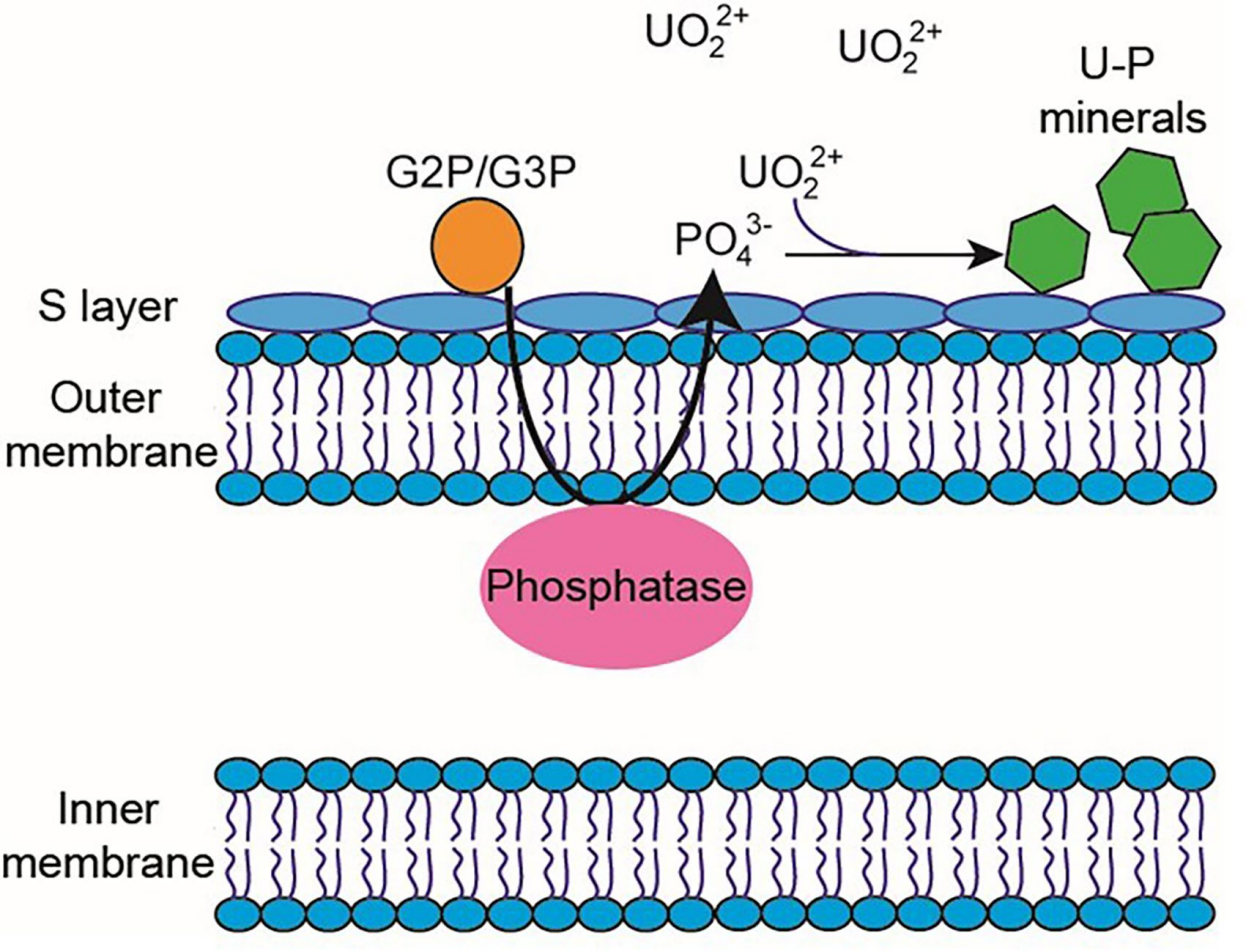
Diagram of the periplasmic phosphatase-mediated biomineralization process. Phosphatase releases inorganic phosphate from an organic phosphate source (e.g., G2P/G3P) to interact with U^VI^ and produce the precipitation of uranium phosphates.

**Figure 9:**
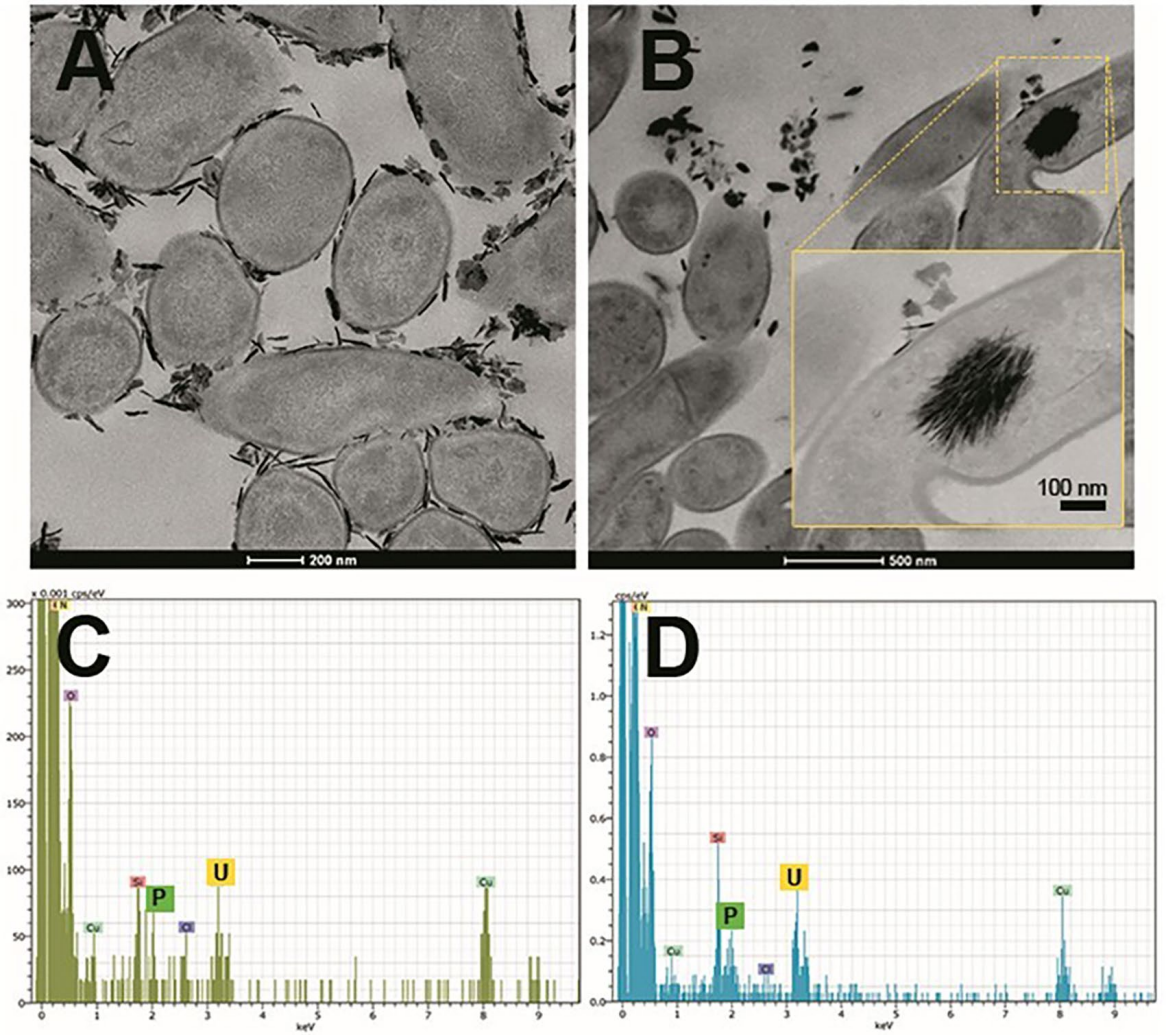
Scanning transmission electron microscopy (STEM) micrographs of thin sections of *Amycolatopsis ruanii*/bacterial consortium (*Bradyrhizobium*-*Rhizobium* and *Pseudomonas*) cells treated with uranium **(A)**, and uranium + glycerol-2-phosphate **(B)**. Extracellular and intracellular precipitates of uranium phosphates and their corresponding EDX spectra with peaks of U and P **(C,D)** respectively. Personal archive of Dr. Merroun; growth conditions detailed in Povedano-Priego et al. (2019).

Microorganism-mediated biomineralization of uranium is a widely used mechanism, which is applied for the remediation of radionuclide contaminated sites. Large numbers of microorganisms are known for their capacity to biomineralize uranium as metautunite-like precipitates using phytase, phosphatases, or complexed with microbe-associated ligands, such as phosphate, carbonate, or hydroxide functional groups (Lin et al., 2023). Thus, U bioprecipitation results frequently from an enzymatic process. Microbial phosphatases are involved in the U biomineralization, since these enzymes release inorganic phosphates (Pi) by hydrolysing organic phosphate substrates, which interact with the radionuclide and precipitate in the form of an insoluble phosphate mineral (Figure 8). A plethora of bacteria isolated from radionuclide and metal contaminated sites have demonstrated uranium precipitation owing their acid or alkaline phosphatase activities. As examples of these, Gram-positive bacteria *Bacillus sphaericus* JG-7B (Merroun et al., 2011), *Paenibacillus* sp. JG-TB8 (facultative anaerobic bacterium; Reitz et al., 2014), and *Microbacterium* sp. A9 (Theodorakopoulos et al., 2015) and many Gram-negative members of Alphaproteobacteria, Deltaproteobacteria, and Gammaproteobacteria classes (Kolhe et al., 2018; Pinel-Cabello et al., 2021b) should be mentioned. In most of these cases, U bioprecipitation occurs in the form of a stable and insoluble meta-autunite mineral (uranyl phosphate mineral phase). On the contrary, Beazley et al. (2007) isolated an *Arthrobacter* sp., from sub-surface soils at the US Oak Ridge field Research Centre, as a negative phosphatase bacterium, which was unable to precipitate uranium. Different phosphatase enzymes were identified as responsible for the uranium biomineralization that includes PhoY, the alkaline phosphatase PhoK, and the acid phosphatase PhoN in *Caulobacter crescentus*, in *Sphingomonas*, and *Serratia* sp., respectively (Nilgiriwala et al., 2008; Paterson-Beedle et al., 2012; Yung and Jiao, 2014). Biomineralization by polyphosphates has also been proven to occur in many uranium contaminated sites. Polyphosphates (phosphate polymers) can also be degraded as an alternative to obtain phosphates that precipitate with the metal intracellularly (Acharya et al., 2017). For example, in *Pseudomonas aeruginosa*, an over-expression of the polyphosphate kinase (ppk) gene has been reported to result in the release of phosphates, which precipitate in the form of uranyl phosphate minerals in the cell membranes (Renninger et al., 2004). *Pseudomonas* has been identified in several bentonite samples (Lopez-Fernandez et al., 2015; Povedano-Priego et al., 2021), including uranium-treated microcosms in the presence of G2P (Povedano-Priego et al., 2022). G2P as an organic phosphate source enrich *Pseudomonas* since these bacteria have been described to possess gene encoding phosphatases that may have the G2P as a substrate to release inorganic phosphate (Liu et al., 2016; Sarikhani et al., 2019). Other environmental microorganisms such as *Bacillus*, *Rhanella*, *Arthrobacter* and *Cellulomonas* have shown their capacity to immobilize U as biogenic uranyl phosphate minerals owing to their polyphosphate metabolism or organophosphate hydrolase activity (Martinez et al., 2007; Sivaswamy et al., 2011). Povedano-Priego et al. (2019) reported the phosphatase activity in *Amycolatopsis ruanii*, a significantly abundant genus in G2P-uranium treated bentonites. The highest concentrations of P_i_ in solution were observed when *Amycolatopsis* was treated with G2P. However, electron-dense precipitates were found with, and without the G2P amendment (Figures 9A,B). These precipitates were composed of phosphorus and uranium (Figures 9C,D). Thus, the biomineralization of uranium has also been observed without the G2P amendment (Figure 9A). Uranium phosphates have been identified extracellularly and at the cell-wall level with and without G2P (Figures 9A,B), while intracellular needle-like fibril precipitates were only detected in presence of G2P (Figure 9B). These results confirm the capacity of these bacteria to biomineralize uranium, which had been enhanced by the G2P amendment through phosphatase activity. In the study of Martinez-Rodriguez et al. (2022) *Microbacterium* sp. Be9 strain was used, previously isolated from U-mill tailings and determined that the U-biomineralization process was dependent on the type of phosphate source. This is relevant for the bioremediation of uranium since the solubilization of orthophosphates derived from waste products containing P-compounds could occur (Martinez-Rodriguez et al., 2022). However, high concentrations of uranium could produce a harmful effect on bacterial activity. To avoid this negative effect, Sánchez-Castro et al. (2021) embedded bacterial cells of *Stenotrophomonas* sp. Br8 in an alginate matrix to protect the cells from hazardous agents and enhanced the immobilization rate. This methodology could be applied to bioremediate U-contaminated mining water.

Nonetheless, U-phosphate biomineralization is affected by many environmental factors including temperature, carbonate, pH, and ammonium. In alkaline conditions, for example, carbonate impacts U-phosphate biomineralization due to the higher affinity of CO_3_^2−^ with UO_2_^2+^ than PO_4_^3−^. Additionally, NH_4_^+^ can interact with UO_2_^2+^ as well as with PO_4_^3−^ promoting the precipitation of uranium. A handful of studies have profiled the influence of uranium on the microbial communities of several ecosystems, which is known to induce changes in functional processes as well as in the taxonomic composition (Suriya et al., 2017; Sutcliffe et al., 2017; Sánchez-Castro et al., 2020). The microbial communities in different U-treated bentonites have been largely studied by using multiple state-of-the-art techniques (Lopez-Fernandez et al. 2018b; Povedano-Priego et al., 2019). Anoxic conditions will prevail in such systems and the presence of strict and facultative anaerobes is well established (Povedano-Priego et al., 2022).

## 6. Discussion and future perspectives

This review highlights the potential role of microorganisms related to the safety of DGRs. As previously mentioned, this type of disposal is based on a multi-barrier system in which the waste will be encapsulated in corrosion-resistant metal canisters, surrounded by filling and sealing materials, which will be placed at depths of between 500–1,000 m in a stable geological formation. Microorganisms are able to colonize almost every single habitat on Earth, and the barriers of the DGRs are no exception. Even though microorganisms could be passively introduced through human manipulation of the different materials during the construction of the DGR, natural microbial communities in the different barriers may be the most important source of microbial activity within the repository. It is worth noting the importance of microorganisms naturally occurring in the filling and sealing materials (e.g., bentonite clay) that will be in direct contact with the metal canisters and, therefore, close to the radioactive waste. For this reason, the different bentonites (e.g., Opalinus, Boom and Spanish clay) have been extensively studied at microbiological level in order to understand the different processes in which microorganisms may be involved.

As we have tried to show in this review, microorganisms could influence the stability and security of the DGRs at different levels. Certain types of microbial communities (e.g., SRB) can cause the alteration of metal canisters (e.g., steel-based materials and copper) through direct or indirect processes, also known as microbially influenced corrosion (MIC). The canister corrosion, in the worst-case scenario, could suffer small pits or fissures, leaving the residue exposed to the next engineered barrier (the filling and sealing material). Moreover, MIC could be involved in the gas production in the DGR environment. MIC, radiolysis of water and microbial metabolism could promote a rise in the gas phase in the DGRs and produce an increase in pressure which would affect the integrity of the clay barrier. However, the metabolism of microorganisms could have a double effect related to gases in the repositories. The production of certain gases due to the metabolism of some bacterial groups may be used by other groups for their own metabolism, thus minimizing the possible pressure produced, positively affecting the safety of the DGR.

Additionally, microbial activity can indirectly influence solubility and hence the mobilization of radionuclides by the alteration of the geochemical conditions within the DGRs. These changes may result in changes in their oxidation state, affecting their solubility and mobility through the different barriers. Nonetheless, microorganisms could be involved in the mobility of these radionuclides. As mentioned before, several mechanisms such as biotransformation, biosorption, bioaccumulation, and biomineralization, may be involved in the interaction microbe-radionuclides. The result of these interactions produces a positive effect since certain microorganisms are able to immobilize elements such as Se, Eu, Cm, and U, making them unavailable to other organisms or, preventing their diffusion through the subsequent engineered barriers and then through to the environment.

Notwithstanding, the purpose of this review is to give an overview of the effect that microorganisms could have on the stability and safety of future DGRs. It will be necessary to consider different factors that could affect the bacterial communities present in the bentonite after the definitive closure of a DGR. One of these parameters is the temperature evolution throughout the confinement period. This temperature will initially increase due to the heat resulting from the decay of the HLW, and then slowly decrease until reaching environmental geosphere conditions over a period of 100,000 years (McMurry et al., 2003). Most models suggest a maximum temperature of 80°C–100°C reached within the first 100 years (McMurry et al., 2003; Butov et al., 2017; Leong et al., 2021). Moreover, another aspect to consider is the compaction density of the bentonite. The compaction has been shown to affect SRB viability demonstrating that the higher the density is, the lower the viability of this bacterial group (Bengtsson and Pedersen, 2017). In addition to these determinant factors, others must be considered such as the evolution to an anaerobic environment due to the gradual consumption of oxygen by the organisms, the slow diffusion of pore water through the bentonite and the low concentration of organic matter, among others. All these factors make the DGR system a harsh environment for certain groups of microorganisms preventing their growth and even their survival. Otherwise, many bacteria could remain in a dormant state until growth conditions became favorable again, such as size decrease, spore formation or cell desiccation (O'Sullivan et al., 2015). For this reason, it is crucial to investigate the microbial influence and the conditions that affect the bacterial communities within the DGR environment, in order to ensure the safety of the repository facilities throughout their service life.

## Author contributions

MAR-F and MLM planned the work. MAR-F wrote the first draft of the manuscript, which was revised by MLM. MFM-M, CP-P, MM-H, and FJ assisted writing some sections of the manuscript. The final version was edited and revised by MAR-F, MFM-M, CP-P, MM-H, FJ, and MLM. All authors contributed to the article and approved the submitted version.

## Funding

This work is funded by FEDER/Junta de Andalucía-Consejería de Transformación Económica, Industria, Conocimiento, y Universidades/Proyecto (B-RNM-250-UGR20) and grant RTI2018-101548-B-I00 of MCIN/AEI/10.13039/501100011033/ FEDER “Una manera de hacer Europa. B.RNM.250.UGR20 financiado por la Consejería de Universidad, Investigación e Innovación de la Junta de Andalucía y por FEDER.”

## Conflict of interest

The authors declare that the research was conducted in the absence of any commercial or financial relationships that could be construed as a potential conflict of interest.

## Publisher’s note

All claims expressed in this article are solely those of the authors and do not necessarily represent those of their affiliated organizations, or those of the publisher, the editors and the reviewers. Any product that may be evaluated in this article, or claim that may be made by its manufacturer, is not guaranteed or endorsed by the publisher.

## References

[ref1] Abdalla Z. A. Y. Ismail M. Y. A. Njoroge E. G. Hlatshwayo T. T. Wendler E. Malherbe J. B. (2020). Migration behaviour of selenium implanted into polycrystalline 3C–SiC. Vacuum 175:109235. doi: 10.1016/j.vacuum.2020.109235

[ref2] Abdullahi S. L. Audu A. A. (2017). Comparative analysis on chemical composition of bentonite clays obtained from Ashaka and tango deposits in Gombe state, Nigeria. ChemSearch J. 8, 35–40.

[ref3] Abrahamsen L. Arnold T. Brinkmann H. Leys N. Merroun M. Mijnendonckx K.. (2015). A review of anthropogenic organic wastes and their degradation behaviour, Microbiology In Nuclear waste Disposal (MIND Project). Available at: https://igdtp.eu/wpcontent/uploads/2017/10/MIND-2015-12-D1.1-ReviewOfAnthropogenicOrganicWastes.pdf

[ref4] Abramova E. Popova N. Artemiev G. Boldyrev K. Kazakov K. Kryuchkov D.. (2023). Biological factors affecting the evolution of safety barrier materials in the Yeniseisky deep geological repository. Eng. Geol. 312:106931. doi: 10.1016/j.enggeo.2022.106931

[ref5] Acharya C. Chandwadkar P. Nayak C. (2017). Unusual versatility of the filamentous, Diazotrophic cyanobacterium *Anabaena torulosa* revealed for its survival during prolonged uranium exposure. Appl. Environ. Microbiol. 83, e03356–e03316. doi: 10.1128/AEM.03356-16, PMID: 28258135 PMC5394331

[ref7] Ahonen L. Pedersen K. Small J. Mijnendonckx K. Weetjens E. Wouters K. (2016). Year one evaluation report, microbiology in nuclear waste disposal (MIND project). Available at: https://mind15.eu/wp-content/uploads/2015/11/MIND-Deliverable-3.1-Y1-Evaluation.pdf

[ref8] Akob D. Lee S. Sheth M. Küsel K. Watson D. Palumbo A. V.. (2012). Gene expression correlates with process rates quantified for sulfate- and Fe(III)-reducing bacteria in U^VI^-contaminated sediments. Front. Microbiol. 3:280. doi: 10.3389/fmicb.2012.00280, PMID: 22908009 PMC3415069

[ref9] Alwaeli M. Mannheim V. (2022). Investigation into the current state of nuclear energy and nuclear waste management—a state-of-the-art review. Energies 15:4275. doi: 10.3390/en15124275

[ref10] Ansoborlo E. Bion L. Doizi D. Moulin C. Lourenco V. Madic C.. (2007). Current and future radionuclide speciation studies in biological media. Radiat. Prot. Dosimetry 127, 97–102. doi: 10.1093/rpd/ncm258, PMID: 17578878

[ref11] Atwood D. A. (2010). Radionuclides in the environment (EIC books). Weinheim: WILEY.

[ref12] Ayangbenro A. S. Babalola O. O. (2017). A new strategy for heavy metal polluted environments: a review of microbial biosorbents. Int. J. Environ. Res. Public Health 14:94. doi: 10.3390/ijerph14010094, PMID: 28106848 PMC5295344

[ref13] Bachran M. Kluge S. Lopez-Fernandez M. Cherkouk A. (2018). Microbial diversity in an arid, naturally saline environment. Microb. Ecol. 78, 494–505. doi: 10.1007/s00248-018-1301-2 30593603

[ref14] Bader M. Moll H. Steudtner R. Lösch H. Drobot B. Stumpf T.. (2019). Association of Eu(III) and cm(III) onto an extremely halophilic archaeon. Environ. Sci. Pollut. Res. 26, 9352–9364. doi: 10.1007/s11356-019-04165-7, PMID: 30721439

[ref15] Baggio G. Groves R. A. Chignola R. Piacenza E. Presentato A. Lewis I. A.. (2021). Untargeted metabolomics investigation on selenite reduction to elemental selenium by bacillus mycoides SeITE01. Front. Microbiol. 12:711000. doi: 10.3389/fmicb.2021.711000, PMID: 34603239 PMC8481872

[ref16] Bagnoud A. Chourey K. Hettich R. L. de Bruijn I. Andersson A. F. Leupin O. X.. (2016a). Reconstructing a hydrogen-driven microbial metabolic network in Opalinus clay rock. Nat. Commun. 7:12770. doi: 10.1038/ncomms12770, PMID: 27739431 PMC5067608

[ref17] Bagnoud A. de Bruijn I. Andersson A. F. Diomidis N. Leupin O. X. Schwyn B.. (2016b). A minimalistic microbial food web in an excavated deep subsurface clay rock. FEMS Microbiol. Ecol. 92:fiv138. doi: 10.1093/femsec/fiv138, PMID: 26542073

[ref18] Bagnoud A. Leupin O. Schwyn B. Bernier-Latmani R. (2016c). Rates of microbial hydrogen oxidation and sulfate reduction in Opalinus clay rock. Appl. Geochem. 72, 42–50. doi: 10.1016/J.APGEOCHEM.2016.06.011

[ref19] Barkleit A. Moll H. Bernhard G. (2009). Complexation of uranium(VI) with peptidoglycan. Dalton Trans. 27, 5379–5385. doi: 10.1039/b818702a, PMID: 19565089

[ref20] Beaton D. Pelletier P. Goulet R. R. (2019). Microbial degradation of cellulosic material and gas generation: implications for the management of low-and intermediate-level radioactive waste. Front. Microbiol. 10, 1–13. doi: 10.3389/fmicb.2019.00204, PMID: 30814985 PMC6381020

[ref21] Beazley M. J. Martinez R. J. Sobecky P. A. Webb S. M. Taillefert M. (2007). Uranium biomineralization as a result of bacterial phosphatase activity: insights from bacterial isolates from a contaminated subsurface. Environ. Sci. Technol. 41, 5701–5707. doi: 10.1021/es070567g, PMID: 17874776

[ref22] Ben Ali Gam Z. Thioye A. Cayol J.-L. Joseph M. Fauque G. Labat M. (2018). Characterization of *Desulfovibrio salinus* sp. nov., a slightly halophilic sulfate-reducing bacterium isolated from a saline lake in Tunisia. Int. J. Syst. Evol. Microbiol. 68, 715–720. doi: 10.1099/ijsem.0.002567, PMID: 29458461

[ref23] Bengtsson A. Pedersen K. (2016). Microbial sulfate-reducing activity overload pressure and density in water saturated boom clay. Appl. Clay Sci. 132-133, 542–551. doi: 10.1016/j.clay.2016.08.002

[ref24] Bengtsson A. Pedersen K. (2017). Microbial sulphide-producing activity in water saturated Wyoming MX-80, Asha and Calcigel bentonites at wet densities from 1500 to 2000 kg m− 3. Appl. Clay Sci. 137, 203–212. doi: 10.1016/j.clay.2016.12.024

[ref25] Benko I. Nagy G. Tanczos B. Ungvari E. Sztrik A. Eszenyi P.. (2012). Subacute toxicity of nano-selenium compared to other selenium species in mice. Environ. Toxicol. Chem. 31, 2812–2820. doi: 10.1002/etc.1995, PMID: 22927138

[ref27] Brim H. McFarlan S. C. Fredrickson J. K. Minton K. W. Zhai M. Wackett L. P.. (2000). Engineering *Deinococcus radiodurans* for metal remediation in radioactive mixed waste environments. Nat. Biotechnol. 18, 85–90. doi: 10.1038/71986, PMID: 10625398

[ref28] Brown A. R. Boothman C. Pimblott S. M. Lloyd J. R. (2015). The impact of gamma radiation on sediment microbial processes. Appl. Environ. Microbiol. 81, 4014–4025. doi: 10.1128/AEM.00590-15, PMID: 25841009 PMC4524164

[ref29] Bulgarini A. Lampis S. Turner R. J. Vallini G. (2021). Biomolecular composition of capping layer and stability of biogenic selenium nanoparticles synthesized by five bacterial species. J. Microbial. Biotechnol. 14, 198–212. doi: 10.1111/1751-7915.13666, PMID: 33068075 PMC7888468

[ref30] Burns P. C. (2012). Nuclear Fuel in a Reactor Accident. Science 335, 1184–1189. doi: 10.1126/science.1211285 22403382

[ref31] Burzan N. Murad Lima R. Frutschi M. Janowczyk A. Reddy B. Rance A.. (2022). Growth and persistence of an aerobic microbial Community in Wyoming Bentonite MX-80 despite anoxic in situ conditions. Front. Microbiol. 13:858324. doi: 10.3389/fmicb.2022.858324, PMID: 35547138 PMC9082992

[ref32] Butov R. A. Drobyshevsky N. I. Moiseenko E. V. Tokarev Y. N. (2017). 3D numerical modelling of the thermal state of deep geological nuclear waste repositories. J. Phys 899:052002. doi: 10.1088/1742-6596/899/5/052002

[ref33] Castañeda-Carrión I. N. Sheik C. S. Krumholz L. R. (2010). *Desulfovibrio africanus* subsp. uniflagellum subsp. nov., a sulfate-reducing bacterium from a uranium-contaminated subsurface aquifer. Int. J. Syst. Evol. Microbiol. 60, 880–886. doi: 10.1099/ijs.0.006668-0 19661495

[ref34] Černoušek T. Shrestha R. Kovářová H. Špánek R. Ševců A. Sihelská K.. (2019). Microbially influenced corrosion of carbon steel in the presence of anaerobic sulfate-reducing bacteria. Corros. Engineer. Sci. Technol. 55, 127–137. doi: 10.1080/1478422X.2019.170064

[ref36] Challenger F. (1945). Biological methylation. Chem. Rev. 36, 315–361. doi: 10.1021/cr60115a003, PMID: 36852676

[ref37] Chandwadkar P. Misra H. S. Acharya C. (2018). Uranium biomineralization induced by a metal tolerant: *Serratia* strain under acid, alkaline and irradiated conditions. Metallomics 10, 1078–1088. doi: 10.1039/C8MT00061A, PMID: 29999065

[ref38] Chasteen T. G. Bentley R. (2003). Biomethylation of selenium and tellurium: microorganisms and plants. Chem. Rev. 103, 1–26. doi: 10.1021/cr010210+, PMID: 12517179

[ref39] Chen X. Ottosen L. D. M. Kofoed M. V. W. (2019). How low can You go: methane production of *Methanobacterium congolense* at low CO2 concentrations. Front. Bioeng. Biotechnol. 7:34. doi: 10.3389/fbioe.2019.00034 30899758 PMC6416169

[ref40] Chen J. Qin Z. Shoesmith D. W. (2011). Long-term corrosion of copper in a dilute anaerobic sulfide solution. Electrochim. Acta 56, 7854–7861. doi: 10.1016/j.electacta.2011.04.086

[ref41] Chen S. Wang P. Zhang D. (2014). Corrosion behaviour of copper under biofilm of sulfate-reducing bacteria. Corros. Sci. 87, 407–415. doi: 10.1016/j.corsci.2014.07.001

[ref42] Choudhary S. Sar P. (2009). Characterization of a metal resistant *pseudomonas* sp. isolated from uranium mine for its potential in heavy metal (Ni2+, Co2+, Cu2+, and Cd2+) sequestration. Bioresour. Technol. 100, 2482–2492. doi: 10.1016/j.biortech.2008.12.015, PMID: 19162475

[ref43] Close D. M. Nelson W. H. Bernhard W. A. (2013). DNA damage by the direct effect of ionizing radiation: products produced by two sequential one-electron oxidations. J. Phys. Chem. A 117, 12608–12615. doi: 10.1021/jp4084844, PMID: 24131335

[ref44] Cologgi D. L. Speers A. M. Bullard B. A. Kelly S. D. Reguera G. (2014). Enhanced uranium immobilization and reduction by *Geobacter sulfurreducens* biofilms. Appl. Environ. Microbiol. 80, 6638–6646. doi: 10.1128/AEM.02289-14, PMID: 25128347 PMC4249037

[ref45] Davis T. Z. Stegelmeier B. L. Welch K. D. Pfister J. A. Panter K. E. Hall J. O. (2013). Comparative oral dose toxicokinetics of selenium compounds commonly found in selenium accumulator plants. J. Anim. Sci. 91, 4501–4509. doi: 10.2527/jas.2012-6101, PMID: 23825349

[ref47] Diep P. Mahadevan R. Yakunin A. F. (2018). Heavy metal removal by bioaccumulation using genetically engineered microorganisms. Front. Bioeng. Biotechnol. 6:157. doi: 10.3389/fbioe.2018.00157, PMID: 30420950 PMC6215804

[ref48] Dobias J. Suvorova E. I. Bernier-Latmani R. (2011). Role of proteins in controlling selenium nanoparticle size. Nanotechnology 22:195605. doi: 10.1088/0957-4484/22/19/195605, PMID: 21430311

[ref49] Dobrowolski R. Szcześ A. Czemierska M. Jarosz-Wikołazka A. (2017). Studies of cadmium(II), lead(II), nickel(II), cobalt(II) and chromium(VI) sorption on extracellular polymeric substances produced by *Rhodococcus opacus* and *Rhodococcus rhodochrous*. Bioresour. Technol. 225, 113–120. doi: 10.1016/j.biortech.2016.11.040, PMID: 27888727

[ref50] Dong H. Jaisi D. P. Kim J. Zhang G. (2009). Microbe-clay mineral interactions. Am. Mineral. 94, 1505–1519. doi: 10.2138/am.2009.3246, PMID: 35688243

[ref51] Dou W. Pu Y. Han X. Song Y. Chen S. Gu T. (2020). Corrosion of cu by a sulfate reducing bacterium in anaerobic vials with different headspace volumes. Bioelectrochemistry 133:107478. doi: 10.1016/j.bioelechem.2020.107478, PMID: 32036296

[ref53] Duro L. Domènech C. Grivé M. Roman-Ross G. Bruno J. Källström K. (2014). Assessment of the evolution of the redox conditions in a low and intermediate level nuclear waste repository (SFR1, Sweden). Appl. Geochem. 49, 192–205. doi: 10.1016/j.apgeochem.2014.04.015

[ref54] El Hajj H. Abdelouas A. Grambow B. Martin C. Dion M. (2010). Microbial corrosion of P235GH steel under geological conditions. Phys. Chem. Earth 35, 248–253. doi: 10.1016/j.pce.2010.04.007

[ref55] Enning D. Garrelfs J. (2014). Corrosion of iron by sulfate-reducing bacteria: new views of an old problem. Appl. Environ. Microbiol. 80, 1226–1236. doi: 10.1128/AEM.02848-13, PMID: 24317078 PMC3911074

[ref56] Eswayah A. S. Smith T. J. Gardiner P. H. E. (2016). Microbial transformations of selenium species of relevance to bioremediation. Appl. Environ. Microbiol. 82, 4848–4859. doi: 10.1128/AEM.00877-16 27260359 PMC4968552

[ref57] Eswayah A. S. Smith T. J. Scheinost A. C. Hondow N. Gardiner P. H. E. (2017). Microbial transformations of selenite by methane-oxidizing bacteria. Appl. Microbiol. Biotechnol. 101, 6713–6724. doi: 10.1007/s00253-017-8380-8, PMID: 28646447 PMC5554269

[ref58] Fakhar A. Gul B. Gurmani A. R. Khan S. M. Ali S. Sultan T.. (2022). Heavy metal remediation and resistance mechanism of *Aeromonas*, *bacillus*, and *pseudomonas*: a review. Crit. Rev. Environ. Sci. Technol. 52, 1868–1914. doi: 10.1080/10643389.2020.1863112

[ref59] Favorito J. E. Grossl P. R. Davis T. Z. Eick M. J. Hankes N. (2021). Soil-plant-animal relationships and geochemistry of selenium in the Western phosphate resource area (United States): a review. Chemosphere 266:128959. doi: 10.1016/j.chemosphere.2020.128959, PMID: 33279237

[ref60] Faybishenko B. Birkholzer J. Sassani D. Swift P. (2017). In: *International approaches for deep geological disposal of nuclear waste: geological challenges in radioactive waste isolation-5th worldwide review*. LBNL-1006984-2016.

[ref61] Fomina M. Gadd G. M. (2014). Biosorption: current perspectives on concept, definition and application. Bioresour. Technol. 160, 3–14. doi: 10.1016/j.biortech.2013.12.102, PMID: 24468322

[ref62] Francis A. J. Nancharaiah Y. V. (2015). “9-in situ and ex situ bioremediation of radionuclide-contaminated soils at nuclear and norm sites” in Woodhead publishing series in energy: Environmental remediation and restoration of contaminated nuclear and norm sites. ed. van Velzen L. (Sawston, United Kingdom: Woodhead) 185–236. doi: 10.1016/B978-1-78242-231-0.00009-0

[ref63] Gabani P. Singh O. V. (2013). Radiation-resistant extremophiles and their potential in biotechnology and therapeutics. Appl. Microbiol. Biotechnol. 97, 993–1004. doi: 10.1007/s00253-012-4642-7, PMID: 23271672

[ref64] Gadd G. M. (2004). Microbial influence on metal mobility and application for bioremediation. Geoderma 122, 109–119. doi: 10.1016/j.geoderma.2004.01.002, PMID: 35817192

[ref65] Gadd G. M. (2009). Biosorption: critical review of scientific rationale, environmental importance and significance for pollution treatment. J. Chem. Technol. Biotechnol. 84, 13–28. doi: 10.1002/jctb.1999

[ref66] Gallois N. Alpha-Bazin B. Bremond N. Ortet P. Barakat M. Piette L.. (2022). Discovery and characterization of UipA, a uranium- and iron-binding PepSY protein involved in uranium tolerance by soil bacteria. ISME J. 16, 705–716. doi: 10.1038/s41396-021-01113-7, PMID: 34556817 PMC8857325

[ref67] Gao W. Francis A. J. (2013). Fermentation and hydrogen metabolism affect uranium reduction by clostridia. ISRN Biotechnol. 2013, 1–11. doi: 10.5402/2013/657160, PMID: 25937978 PMC4393052

[ref68] García-Romero E. María Manchado E. Suárez M. García-Rivas J. (2019). Spanish bentonites: a review and new data on their geology, mineralogy, and crystal chemistry. Fortschr. Mineral. 9:696. doi: 10.3390/min9110696

[ref69] Gerber U. Zirnstein I. Krawczyk-Bärsch E. Lünsdorf H. Arnold T. Merroun M. L. (2016). Combined use of flow cytometry and microscopy to study the interactions between the gram-negative betaproteobacterium *Acidovorax facilis* and uranium(VI). J. Hazard. Mater. 317, 127–134. doi: 10.1016/j.jhazmat.2016.05.062, PMID: 27262280

[ref70] Gerstner E. (2009). Nuclear energy: the hybrid returns. Nature 460, 25–28. doi: 10.1038/460025a, PMID: 19571861

[ref71] Gorietti D. Giardina I. Arginelli D. Battisti P. (2017). Determination of plutonium, americium and curium isotopes in radioactive metal wastes deriving from nuclear decommissioning. J. Radioanal. Nucl. Chem. 314, 1785–1792. doi: 10.1007/s10967-017-5553-y

[ref72] Grigoryan A. A. Jalique D. R. Stroes-Gascoyne S. Wolfaardt G. M. Keech P. G. Korber D. R. (2021). Prediction of bacterial functional diversity in clay microcosms. Heliyon 7:e08131. doi: 10.1016/j.heliyon.2021.e08131, PMID: 34703919 PMC8524152

[ref73] Grouzdev D. S. Safonov A. Babich T. L. Tourova T. P. Krutkina M. S. Nazina T. N. (2018). Draft genome sequence of a dissimilatory U(VI)-reducing bacterium, *Shewanella xiamenensis* strain DCB2-1, isolated from nitrate- and radionuclide-contaminated groundwater in Russia. Genome Announc. 6, e00555–e00518. doi: 10.1128/genomeA.00555-18, PMID: 29930062 PMC6013632

[ref74] Guo G. Fall M. (2021). Advances in modelling of hydro-mechanical processes in gas migration within saturated bentonite: a state-of-art review. Eng. Geol. 287:106123. doi: 10.1016/j.enggeo.2021.106123

[ref75] Hall D. S. Behazin M. Jeffrey Binns W. Keech P. G. (2021). An evaluation of corrosion processes affecting copper-coated nuclear waste containers in a deep geological repository. Prog. Mater. Sci. 118:100766. doi: 10.1016/j.pmatsci.2020.100766

[ref76] Hamed M. M. Holiel M. El-Aryan Y. F. (2017). Removal of selenium and iodine radionuclides from waste solutions using synthetic inorganic ion exchanger. J. Mol. Liq. 242, 722–731. doi: 10.1016/j.molliq.2017.07.035

[ref77] Handrlica J. (2019). Reprocessing of nuclear fuel: certain legal issues arising from this unique technology. Int. J. Legal Res. 9, 150–161.

[ref78] Hassan R. S. Abass M. R. Eid M. A. Abdel-Galil E. A. (2021). Sorption of some radionuclides from liquid waste solutions using anionic clay hydrotalcite sorbent. Appl. Radiat. Isot. 178:109985. doi: 10.1016/j.apradiso.2021.109985, PMID: 34678639

[ref79] Hedrich S. Schlömann M. Barrie Johnson D. (2011). The iron-oxidizing proteobacteria. Microbiology 157, 1551–1564. doi: 10.1099/mic.0.045344-0, PMID: 21511765

[ref80] Hökmark H. Fälth B. (2003). Thermal dimensioning of the deep repository. Influence of canister spacing, canister power, rock thermal properties and nearfield design on the maximum canister surface temperature. SKB Technical Report TR-03-09, Sweden.

[ref81] Holmboe M. Norrfors K. K. Jonsson M. Wold S. (2011). Effect of γ-radiation on radionuclide retention in compacted bentonite. Radiat. Phys. Chem. 80, 1371–1377. doi: 10.1016/j.radphyschem.2011.08.004

[ref82] Horvath A. Rachlew E. (2016). Nuclear power in the 21st century: challenges and possibilities. Ambio 45, 38–49. doi: 10.1007/s13280-015-0732-y, PMID: 26667059 PMC4678124

[ref83] Huang W. H. Chen W. C. (2004). Swelling behavior of a potential buffer material under simulated near field environment. J. Nucl. Sci. Technol. 41, 1271–1279. doi: 10.1080/18811248.2004.9726356

[ref84] Hufton J. Harding J. Smith T. Romero-González M. E. (2021). The importance of the bacterial cell wall in uranium(VI) biosorption. Phys. Chem. Chem. Phys. 23, 1566–1576. doi: 10.1039/D0CP04067C, PMID: 33404558

[ref85] Hupert-Kocurek K. Saczyńska A. Piotrowska-Seget Z. (2013). Cadmium increases catechol 2,3-dioxygenase activity in *Variovorax* sp. 12S, a metal-tolerant and phenol-degrading strain. Antonie Van Leeuwenhoek 104, 845–853. doi: 10.1007/s10482-013-9997-y, PMID: 23934429

[ref86] IAEA. (2009a). Classification of radioactive waste. General safety guide [internet]. Vienna, International Atomic Energy Agency. IAEA safety standards series, no. GSG-1 STI/PUB/1419. Available at: https://www-pub.iaea.org/mtcd/publications/pdf/pub1419_web.pdf

[ref87] IAEA. (2009b). Status and trends of nuclear technologies: Report of the international project on innovative nuclear reactors and fuel cycles (INPRO). Vienna, International Atomic Energy Agency. Available at: https://www-pub.iaea.org/MTCD/Publications/PDF/TE_1622_Web.pdf

[ref88] IAEA. (2015). Climate change and nuclear power 2015. Vienna, International Atomic Energy Agency. Available at: https://www-pub.iaea.org/MTCD/Publications/PDF/CCANP2015Web-78834554.pdf

[ref89] IAEA. (2018). Status and trends in spent fuel and radioactive waste management. Vienna, International Atomic Energy Agency. Nuclear Energy Series No. NW-T-1.14, 74. Available at: https://www-pub.iaea.org/MTCD/Publications/PDF/PUB1963_web.pdf

[ref91] Jain R. Jordan N. Tsushima S. Hübner R. Weiss S. Lens P. N. L. (2017). Shape change of biogenic elemental selenium nanomaterials from nanospheres to nanorods decreases their colloidal stability. Environ. Sci. Nano 4, 1054–1063. doi: 10.1039/c7en00145b

[ref92] Jroundi F. Descostes M. Povedano-Priego C. Sánchez-Castro I. Suvannagan V. Grizard P.. (2020). Profiling native aquifer bacteria in a uranium roll-front deposit and their role in biogeochemical cycle dynamics: insights regarding in situ recovery mining. Sci. Total Environ. 721:137758. doi: 10.1016/j.scitotenv.2020.137758, PMID: 32179349

[ref93] Jung K. W. Lim S. Bahn Y. S. (2017). Microbial radiation-resistance mechanisms. J. Microbiol. 55, 499–507. doi: 10.1007/s12275-017-7242-5, PMID: 28664512

[ref94] Junier P. Vecchia E. D. Bernier-Latmani R. (2011). The response of *Desulfotomaculum reducens* MI-1 to U^VI^ exposure: a transcriptomic study. Geomicrobiol J. 28, 483–496. doi: 10.1080/01490451.2010.512031

[ref96] Kamnev A. A. Mamchenkova P. Dyatlova Y. A. Tugarova A. V. (2017). FTIR spectroscopic studies of selenite reduction by cells of the rhizobacterium *Azospirillum brasilense* Sp7 and the formation of selenium nanoparticles. J. Mol. Struct. 1140, 106–112. doi: 10.1016/j.molstruc.2016.12.003

[ref97] Kessi J. Hanselmann K. W. (2004). Similarities between the abiotic reduction of selenite with glutathione and the dissimilatory reaction mediated by *Rhodospirillum rubrum* and *Escherichia coli*. J. Biol. Chem. 279, 50662–50669. doi: 10.1074/jbc.M405887200, PMID: 15371444

[ref98] Kim J. Dong H. Yang K. Park H. Elliott W. C. Spivack A.. (2019). Naturally occurring, microbially induced smectite-to-illite reaction. Geology 47, 535–539. doi: 10.1130/G46122.1

[ref99] Kip N. Van Veen J. A. (2015). The dual role of microbes in corrosion. Int. Soc. Microb. Ecol. J. 9, 542–551. doi: 10.1038/ismej.2014.169, PMID: 25259571 PMC4331587

[ref100] Kolhe N. Zinjarde S. Acharya C. (2018). Responses exhibited by various microbial groups relevant to uranium exposure. Biotechnol. Adv. 36, 1828–1846. doi: 10.1016/j.biotechadv.2018.07.002, PMID: 30017503

[ref101] Kónya J. Nagy N. M. (2018). “Environmental radioactivity” in Nuclear and radiochemistry. eds. Kónya J. Nagy N. M.. 2nd ed (Netherlands: Elsevier), 399–419.

[ref102] Kooyman T. Buiron L. Rimpault G. (2018). A comparison of curium, neptunium and americium transmutation feasibility. Ann. Nucl. Energy 112, 748–758. doi: 10.1016/j.anucene.2017.09.041

[ref103] Kora A. J. (2018). *Bacillus cereus*, selenite-reducing bacterium from contaminated lake of an industrial area: a renewable nanofactory for the synthesis of selenium nanoparticles. Bioresourc. Bioprocess. 5:30. doi: 10.1186/s40643-018-0217-5

[ref104] Kumari I. Kumar B. V. R. Khanna A. (2020). A review on UREX processes for nuclear spent fuel reprocessing. Nucl. Eng. Des. 358:110410. doi: 10.1016/j.nucengdes.2019.110410

[ref105] Kurmakova I. Kupchyk O. Bondar O. Demchenko N. Vorobyova V. (2019). Corrosion of copper in a medium of bacteria sulfate reduction proceeding. J. Chem. Technol. Metallurgy 54, 416–422.

[ref106] Kushwaha S. Marcus A. K. Rittmann B. E. (2018). pH-dependent speciation and hydrogen (H_2_) control U(VI) respiration by *Desulfovibrio vulgaris*. Biotechnol. Bioeng. 115, 1465–1474. doi: 10.1002/bit.26579 29476629

[ref107] Lampis S. Zonaro E. Bertolini C. Cecconi D. Monti F. Micaroni M.. (2017). Selenite biotransformation and detoxification by *Stenotrophomonas maltophilia* SeITE02: novel clues on the route to bacterial biogenesis of selenium nanoparticles. J. Hazard. Mater. 324, 3–14. doi: 10.1016/j.jhazmat.2016.02.035, PMID: 26952084

[ref108] Lelieveld J. Kunkel D. Lawrence M. G. (2012). Global risk of radioactive fallout after major nuclear reactor accidents. Atmos. Chem. Phys. 12, 4245–4258. doi: 10.5194/acp-12-4245-2012, PMID: 30557804

[ref109] Lenz M. Van Aelst A. C. Smit M. Corvini P. F. X. Lens P. N. L. (2009). Biological production of selenium nanoparticles from waste waters. Adv. Mat. Res. 71–73, 721–724. doi: 10.4028/www.scientific.net/AMR.71-73.721

[ref110] Leong J. Ponnambalam K. Binns J. Elkamel A. (2021). Thermally constrained conceptual deep geological repository design under spacing and placing uncertainties. Appl. Sci. 11:11874. doi: 10.3390/app112411874

[ref111] Leupin O. X. Bernier-Latmani R. Bagnoud A. Moors H. Leys N. Wouters K.. (2017). Fifteen years of microbiological investigation in Opalinus clay at the Mont Terri rock laboratory (Switzerland). Swiss J. Geosci. 110, 343–354. doi: 10.1007/s00015-016-0255-y PMC7081829 32214982

[ref112] Li H. Zhang J. Wang T. Luo W. Zhou Q. Jiang G. (2008). Elemental selenium particles at nano-size (Nano-se) are more toxic to Medaka (*Oryzias latipes*) as a consequence of hyper-accumulation of selenium: a comparison with sodium selenite. Aquat. Toxicol. 89, 251–256. doi: 10.1016/j.aquatox.2008.07.008, PMID: 18768225

[ref114] Lin H. Zhou M. Li B. Dong Y. (2023). Mechanisms, application advances and future perspectives of microbial-induced heavy metal precipitation: a review. Int. Biodeter. Biodegr. 178:105544. doi: 10.1016/j.ibiod.2022.105544

[ref115] Liu Y. Hedwig S. Schäffer A. Lenz M. Martinez M. (2021). Sulfur amino acid status controls selenium methylation in pseudomonas tolaasii: identification of a novel metabolite from promiscuous enzyme reactions. Appl. Environ. Microbiol. 87, e00104–e00121. doi: 10.1128/AEM.00104-21 33811024 PMC8174768

[ref116] Liu X. Long D. You H. Yang D. Zhou S. Zhang S.. (2016). Phosphatidylcholine affects the secretion of the alkaline phosphatase PhoA in *pseudomonas* strains. Microbiol. Res. 192, 21–29. doi: 10.1016/j.micres.2016.02.001, PMID: 27664720

[ref117] Lopez-Fernandez M. Jroundi F. Ruiz-Fresneda M. A. Merroun M. L. (2020). Microbial interaction with and tolerance of radionuclides: underlying mechanisms and biotechnological applications. J. Microbial. Biotechnol. 14, 810–828. doi: 10.1111/1751-7915.13718 PMC8085914 33615734

[ref118] Lopez-Fernandez M. Cherkouk A. Vilchez-Vargas R. Jauregui R. Pieper D. Boon N.. (2015). Bacterial diversity in bentonites, engineered barrier for deep geological disposal of radioactive wastes. Microb. Ecol. 70, 922–935. doi: 10.1007/s00248-015-0630-7, PMID: 26024740

[ref119] Lopez-Fernandez M. Fernández-Sanfrancisco O. Moreno-García A. Martín-Sánchez I. Sánchez-Castro I. Merroun M. L. (2014). Microbial communities in bentonite formations and their interactions with uranium. Appl. Geochem. 49, 77–86. doi: 10.1016/j.apgeochem.2014.06.022

[ref120] Lopez-Fernandez M. Matschiavelli N. Merroun M. L. (2021). Bentonite geomicrobiology. Microbiol. Nuclear Waste Disposal 2021, 137–155. doi: 10.1016/B978-0-12-818695-4.00007-1

[ref122] Lopez-Fernandez M. Simone D. Wu X. Soler L. Nilsson E. Holmfeldt K.. (2018a). Metatranscriptomes reveal that all three domains of life are active but are dominated by bacteria in the Fennoscandian crystalline granitic continental deep biosphere. MBio 9, e01792–e01718. doi: 10.1128/mBio.01792-18, PMID: 30459191 PMC6247080

[ref123] Lopez-Fernandez M. Vilchez-Vargas R. Jroundi F. Boon N. Pieper D. Merroun M. L. (2018b). Microbial community changes induced by uranyl nitrate in bentonite clay microcosms. Appl. Clay Sci. 160, 206–216. doi: 10.1016/j.clay.2017.12.034

[ref121] Lopez-Fernandez M. Moll H. Merroun M. L. (2018c). Reversible pH-dependent curium(III) biosorption by the bentonite yeast isolate *Rhodotorula mucilaginosa* BII-R8. J. Hazard. Mater. 370, 156–163. doi: 10.1016/j.jhazmat.2018.06.054 30940356

[ref124] Losi M. E. Frankenberger W. T. Jr. (1998). Microbial oxidation and solubilization of precipitated elemental selenium in soil. J. Environ. Qual. 27, 836–843. doi: 10.2134/jeq1998.00472425002700040018x

[ref125] Luo X. Wang Y. Lan Y. An L. Wang G. Li M.. (2022). Microbial oxidation of organic and elemental selenium to selenite. Sci. Total Environ. 833:155203. doi: 10.1016/j.scitotenv.2022.155203, PMID: 35421462

[ref126] Ma B. Charlet L. Fernandez-Martinez A. Kang M. Madé B. (2018). Review of the retention mechanisms of redox-sensitive radionuclides in multi-barrier systems. Appl. Geochem. 100, 414–431. doi: 10.1016/j.apgeochem.2018.12.001

[ref127] Majumder E. L.-W. Wall J. D. (2017). Uranium bio-transformations: chemical or biological processes? Open J. Inorgan. Chem. 7, 28–60. doi: 10.4236/ojic.2017.72003

[ref128] Maleke M. Valverde A. Vermeulen J. G. Cason E. Gomez-Arias A. Moloantoa K.. (2019). Biomineralization and bioaccumulation of europium by a thermophilic metal resistant bacterium. Front. Microbiol. 10, 1–10. doi: 10.3389/fmicb.2019.00081 30761115 PMC6363818

[ref001] Meleshyn A. (2014). Microbial processes relevant for the long-term performance of high-level radioactive waste repositories in clays. Geological Society. (London: Special Publications) 400, 179–194. doi: 10.1144/SP400.6

[ref129] Martinez R. J. Beazley M. J. Taillefert M. Arakaki A. K. Skolnick J. Sobecky P. A. (2007). Aerobic uranium ^VI^ bioprecipitation by metal-resistant bacteria isolated from radionuclide- and metal-contaminated subsurface soils. Environ. Microbiol. 9, 3122–3133. doi: 10.1111/j.1462-2920.2007.01422.x, PMID: 17991039

[ref130] Martinez-Rodriguez P. Sanchez-Castro I. Ojeda J. J. Abad M. M. Descostes M. Merroun M. L. (2022). Effect of different phosphate sources on uranium biomineralization by the microbacterium sp. Be9 strain: a multidisciplinary approach study. Front. Microbiol. 13:1092184. doi: 10.3389/fmicb.2022.1092184 36699588 PMC9868770

[ref131] Masurat P. Eriksson S. Pedersen K. (2010). Evidence of indigenous Sulphate-reducing bacteria in commercial Wyoming bentonite MX-80. Appl. Clay Sci. 47, 51–57. doi: 10.1016/j.clay.2008.07.002

[ref132] McMurry J. Dixon D. A. Garroni J. D. Ikeda B. M. Stroes-Gascoyne S. Baumgartner P.. (2003). *Evolution of a Canadian deep geologic repository: Base scenario*, report 06819, 01200-100092. Ontario Power Generation, Nuclear Waste Management Division. Toronto Canada.

[ref133] Merroun M. L. Nedelkova M. Ojeda J. J. Reitz T. Fernández M. L. Arias J. M.. (2011). Bio-precipitation of uranium by two bacterial isolates recovered from extreme environments as estimated by potentiometric titration, TEM and X-ray absorption spectroscopic analyses. J. Hazard. Mater. 197, 1–10. doi: 10.1016/j.jhazmat.2011.09.049, PMID: 22019055

[ref134] Merroun M. L. Raff J. Rossberg A. Hennig C. Reich T. Selenska-Pobell S. (2005). Complexation of uranium by cells and S-layer sheets of *Bacillus sphaericus* JG-A12. Appl. Environ. Microbiol. 71, 5532–5543. doi: 10.1128/AEM.71.9.5532-5543.2005, PMID: 16151146 PMC1214696

[ref135] Mijnendonckx K. Bleyen N. van Gompel A. Coninx I. Leys N. (2022). pH and microbial community determine the denitrifying activity in the presence of nitrate-containing radioactive waste. Front. Microbiol. 13:968220. doi: 10.3389/fmicb.2022.968220, PMID: 36338040 PMC9634998

[ref136] Mijnendonckx K. Miroslav H. Wang L. Jacops E. Provoost A. Mysara M.. (2019). An active microbial community in boom clay pore water collected from piezometers impedes validating predictive modelling of ongoing geochemical processes. Appl. Geochem. 106, 149–160. doi: 10.1016/j.apgeochem.2019.05.009

[ref137] Mijnendonckx K. Monsieurs P. Černá K. Hlaváčková V. Steinová J. Burzan N.. (2021). Chapter 4: molecular techniques for understanding microbial abundance and activity in clay barriers used for geodisposal. In: Lloyd J. L. Cherkouk A. (Eds.) The microbiology of nuclear waste disposal, (Amsterdam, The Netherlands: Elsevier) 71–96. doi: 10.1016/B978-0-12-818695-4.00004-6

[ref138] Mills M. M. Sanchez A. C. Boisvert L. Payne C. B. Ho T. A. Wang Y. (2022). Understanding smectite to illite transformation at elevated (> 100° C) temperature: effects of liquid/solid ratio, interlayer cation, solution chemistry and reaction time. Chem. Geol. 615:121214. doi: 10.1016/j.chemgeo.2022.121214

[ref139] Mishra A. Malik A. (2013). Recent advances in microbial metal bioaccumulation. Crit. Rev. Environ. Sci. Technol. 43, 1162–1222. doi: 10.1080/10934529.2011.627044, PMID: 35934468

[ref140] Mitchell N. Pérez-Sánchez D. Thorne M. C. (2013). A review of the behaviour of U-238 series radionuclides in soils and plants. J. Radiol. Prot. 33:R17. doi: 10.1088/0952-4746/33/2/R17, PMID: 23612607

[ref141] Mohapatra D. P. Robinson K. A. Huang F. Kirpalani D. Loewen M. C. (2022). Insights into increasing Selenate reductase enzyme activity in the presence of nitrogen-doped graphite electrodes for selenium effluent treatment. Water 14:931. doi: 10.3390/w14060931

[ref142] Moll H. Barkleit A. Frost L. Raff J. (2021). Curium(III) speciation in the presence of microbial cell wall components. Ecotoxicol. Environ. Saf. 227:112887. doi: 10.1016/j.ecoenv.2021.112887, PMID: 34649137

[ref143] Moll H. Lütke L. Bachvarova V. Cherkouk A. Selenska-Pobell S. Bernhard G. (2014). Interactions of the Mont Terri Opalinus clay isolate *Sporomusa* sp. MT-2.99 with curium(III) and europium(III). Geomicrobiol J. 31, 682–696. doi: 10.1080/01490451.2014.889975

[ref144] Moll H. Lütke L. Barkleit A. Bernhard G. (2013). Curium(III) speciation studies with cells of a groundwater strain of *Pseudomonas fluorescens*. Geomicrobiol J. 30, 337–346. doi: 10.1080/01490451.2012.688927

[ref146] NEA. (2020a). Management and disposal of high-level radioactive waste: Global Progress and solutions, OECD Publishing, Paris Available at: https://www.oecd-nea.org/jcms/pl_32567/management-and-disposal-of-high-level-radioactive-waste-global-progress-and-solutions

[ref147] NEA. (2020b). The environmental and ethical basis of geological disposal of Long-lived radioactive wastes, OECD Publishing, Paris. Available at: https://oecd-nea.org/jcms/pl_34631/the-environmental-and-ethical-basis-of-geological-disposal-of-long-lived-radioactive-wastes

[ref148] Newman R. C. Rumash K. Webster B. J. (1992). The effect of pre-corrosion on the corrosion rate of steel in neutral solutions containing sulphide: relevance to microbially influenced corrosion. Corros. Sci. 33, 1877–1884. doi: 10.1016/0010-938X(92)90190-E

[ref149] Newsome L. Morris K. Trivedi D. Atherton N. Lloyd J. R. (2014). Microbial reduction of uranium^VI^ in sediments of different lithologies collected from Sellafield. Appl. Geochem. 51, 55–64. doi: 10.1016/j.apgeochem.2014.09.008

[ref150] Nilgiriwala K. S. Alahari A. Rao A. S. Apte S. K. (2008). Cloning and overexpression of alkaline phosphatase PhoK from *Sphingomonas* sp. strain BSAR-1 for bioprecipitation of uranium from alkaline solutions. Appl. Environ. Microbiol. 74, 5516–5523. doi: 10.1128/AEM.00107-08, PMID: 18641147 PMC2546639

[ref151] Nuclear Waste Management Organization. (2022). Programs around the world for managing used nuclear fuel. Toronto: nwmo; [updated April 2022]. Available at: https://www.nwmo.ca/~/media/Site/Files/PDFs/2022/05/31/12/28/Programs-around-the-world-backgrounder-2022.ashx?la=en

[ref152] O'Sullivan L. A. Roussel E. G. Weightman A. J. Webster G. Hubert C. R. Bell E.. (2015). Survival of *Desulfotomaculum* spores from estuarine sediments after serial autoclaving and high-temperature exposure. ISME J. 9, 922–933. doi: 10.1038/ismej.2014.190, PMID: 25325382 PMC4817712

[ref153] Parihar L. Johal J. K. Singh V. (2013). Bioremediation of uranium in contaminated water samples of Bathinda, Punjab by *Desulfovibrio* genus. J. Soil Sci. Environ. Manage. 4, 1–5. doi: 10.5897/JSSEM12.058

[ref154] Park D. M. Jiao Y. (2014). Modulation of medium pH by *Caulobacter crescentus* facilitates recovery from uranium-induced growth arrest. Appl. Environ. Microbiol. 80, 5680–5688. doi: 10.1128/AEM.01294-14, PMID: 25002429 PMC4178613

[ref155] Paterson-Beedle M. Jeong B. C. Lee C. H. Jee K. Y. Kim W. H. Renshaw J. C.. (2012). Radiotolerance of phosphatases of a *Serratia* sp.: potential for the use of this organism in the biomineralization of wastes containing radionuclides. Biotechnol. Bioeng. 109, 1937–1946. doi: 10.1002/bit.24467, PMID: 22422344

[ref156] Payne R. B. Gentry D. M. Rapp-Giles B. J. Casalot L. Wall J. D. (2002). Uranium reduction by *Desulfovibrio desulfuricans* strain G20 and a cytochrome c3 mutant. Appl. Environ. Microbiol. 68, 3129–3132. doi: 10.1128/AEM.68.6.3129-3132.2002, PMID: 12039777 PMC123926

[ref157] Pedersen K. (2010). Analysis of copper corrosion in compacted bentonite clay as a function of clay density and growth conditions for sulfate-reducing bacteria. J. Appl. Microbiol. 108, 1094–1104. doi: 10.1111/j.1365-2672.2009.04629.x, PMID: 20015208

[ref158] Pedersen K. (2013). Metabolic activity of subterranean microbial communities in deep granitic groundwater supplemented with methane and H_2_. ISME J. 7, 839–849. doi: 10.1038/ismej.2012.144, PMID: 23235288 PMC3603388

[ref160] Periyakaruppan A. Kumar F. Sarkar S. Sharma C. S. Ramesh G. T. (2007). Uranium induces oxidative stress in lung epithelial cells. Arch. Toxicol. 81, 389–395. doi: 10.1007/s00204-006-0167-0, PMID: 17124605 PMC2740373

[ref161] Pinel-Cabello M. Chapon V. Ruiz-Fresneda M. A. Alpha-Bazin B. Berthomieu C. Armengaud J.. (2021a). Delineation of cellular stages and identification of key proteins for reduction and biotransformation of se(IV) by *Stenotrophomonas bentonitica* BII-R7. J. Hazard. Mater. 418:126150. doi: 10.1016/j.jhazmat.2021.126150 34111750

[ref162] Pinel-Cabello M. Jroundi F. López-Fernández M. Geffers R. Jarek M. Jauregui R.. (2021b). Multisystem combined uranium resistance mechanisms and bioremediation potential of *Stenotrophomonas bentonitica* BII-R7: transcriptomics and microscopic study. J. Hazard. Mater. 403:123858. doi: 10.1016/j.jhazmat.2020.123858, PMID: 33264934

[ref163] Plötze M. Kahr G. Stengele R. H. (2003). Alteration of clay minerals — gamma-irradiation effects on physico chemical properties. Appl. Clay Sci. 23, 195–202. doi: 10.1016/S0169-1317(03)00103-0

[ref164] Porcelli D. (2018). “Radioactivity” in Encyclopedia of geochemistry: A comprehensive reference source on the chemistry of the earth. ed. White W. M. (Cham: Springer International Publishing), 1295–1298.

[ref165] Povedano-Priego C. Jroundi F. Lopez-Fernandez M. Morales-Hidalgo M. Martin-Sánchez I. Huertas F. J.. (2022). Impact of anoxic conditions, uranium^VI^ and organic phosphate substrate on the biogeochemical potential of the indigenous bacterial community of bentonite. Appl. Clay Sci. 216:106331. doi: 10.1016/j.clay.2021.106331

[ref166] Povedano-Priego C. Jroundi F. Lopez-Fernandez M. Sánchez-Castro I. Martin-Sánchez I. Huertas F. J.. (2019). Shifts in bentonite bacterial community and mineralogy in response to uranium and glycerol-2-phosphate exposure. Sci. Total Environ. 692, 219–232. doi: 10.1016/j.scitotenv.2019.07.228, PMID: 31349163

[ref167] Povedano-Priego C. Jroundi F. Lopez-Fernandez M. Shrestha R. Spanek R. Martín-Sánchez I.. (2021). Deciphering indigenous bacteria in compacted bentonite through a novel and efficient DNA extraction method: insights into biogeochemical processes within the deep geological disposal of nuclear waste concept. J. Hazard. Mater. 408:124600. doi: 10.1016/j.jhazmat.2020.124600, PMID: 33339698

[ref168] Povedano-Priego C. Jroundi F. Solari P. L. Guerra-Tschuschke I. Abad-Ortega M. D. M. Link A.. (2023). Unlocking the bentonite microbial diversity and its implications in selenium bioreduction and biotransformation: advances in deep geological repositories. J. Hazard. Mater. 445:130557. doi: 10.1016/j.jhazmat.2022.130557, PMID: 36502723

[ref169] Prakash D. Gabani P. Chandel A. K. Ronen Z. Singh O. (2013). Bioremediation: a genuine technology to remediate radionuclides from the environment. J. Microbial. Biotechnol. 6, 349–360. doi: 10.1111/1751-7915.12059, PMID: 23617701 PMC3917470

[ref170] Prăvălie R. Bandoc G. (2018). Nuclear energy: between global electricity demand, worldwide decarbonisation imperativeness, and planetary environmental implications. J. Environ. Manage. 209, 81–92. doi: 10.1016/j.jenvman.2017.12.043, PMID: 29287177

[ref171] Priest N. D. (2001). Toxicity of depleted uranium. Lancet 357, 244–246. doi: 10.1016/S0140-6736(00)03605-9, PMID: 11214120

[ref172] Pushkareva R. Kalinichenko E. Lytovchenko A. Pushkarev A. Kadochnikov V. Plastynina M. (2002). Irradiation effect on physico-chemical properties of clay minerals. Appl. Clay Sci. 21, 117–123. doi: 10.1016/S0169-1317(01)00097-7

[ref173] Rashwan T. L. Asad M. A. Molnar I. L. Behazin M. Keech P. G. Krol M. M. (2022). Exploring the governing transport mechanisms of corrosive agents in a Canadian deep geological repository. Sci. Total Environ. 828:153944. doi: 10.1016/j.scitotenv.2022.153944, PMID: 35192826

[ref174] Rauschenbach I. Posternak V. Cantarella P. McConnell J. Starovoytov V. Häggblom M. M. (2013). *Seleniivibrio woodruffii* gen. Nov., sp. nov., a selenate- and arsenate-respiring bacterium in the *Deferribacteraceae*. Int. J. Syst. Evol. Microbiol. 63, 3659–3665. doi: 10.1099/ijs.0.043547-0, PMID: 23625257

[ref175] Reitz T. Rossberg A. Barkleit A. Selenska-Pobell S. Merroun M. L. (2014). Decrease of U^VI^ immobilization capability of the facultative anaerobic strain *Paenibacillus* sp. JG-TB8 under anoxic conditions due to strongly reduced phosphatase activity. PLoS One 9:e102447. doi: 10.1371/journal.pone.0102447, PMID: 25157416 PMC4144796

[ref176] Renninger N. Knopp R. Nitsche H. Clark D. S. Keasling J. D. (2004). Uranyl precipitation by *Pseudomonas aeruginosa* via controlled polyphosphate metabolism. Appl. Environ. Microbiol. 70, 7404–7412. doi: 10.1128/AEM.70.12.7404-7412.2004, PMID: 15574942 PMC535141

[ref177] Rogiers T. Houdt R. V. Williamson A. Leys N. Boon N. Mijnendonckx K. (2022). Molecular mechanisms underlying bacterial uranium resistance. Front. Microbiol. 13:822197. doi: 10.3389/fmicb.2022.822197, PMID: 35359714 PMC8963506

[ref178] Roh C. Kang C. K. Lloyd J. R. (2015). Microbial bioremediation processes for radioactive waste. Korean J. Chem. Eng. 32, 1720–1726. doi: 10.1007/s11814-015-0128-5, PMID: 35868082

[ref179] Rosa E. A. Tuler S. P. Fischhoff B. Webler T. Friedman S. M. Sclove R. E.. (2010). Nuclear waste: knowledge waste? Science 329, 762–763. doi: 10.1126/science.1193205, PMID: 20705835

[ref180] Rui X. Kwon M. J. O’Loughlin E. J. Dunham-Cheatham S. Fein J. B. Bunker B.. (2013). Bioreduction of hydrogen uranyl phosphate: mechanisms and U(IV) products. Environ. Sci. Technol. 47, 5668–5678. doi: 10.1021/es305258p, PMID: 23634690

[ref181] Ruiz Fresneda M. A. Delgado Martín J. Gómez Bolívar J. Fernández Cantos M. Bosch-Estévez G. Martínez Moreno M. F.. (2018). Green synthesis and biotransformation of amorphous se nanospheres to trigonal 1D se nanostructures: impact on se mobility within the concept of radioactive waste disposal. Environ. Sci. Nano 5, 2103–2116. doi: 10.1039/c8en00221e

[ref182] Ruiz-Fresneda M. A. Eswayah A. S. Romero-González M. Gardiner P. H. E. Solari P. L. Merroun M. L. (2020a). Chemical and structural characterization of SeIV biotransformations by *Stenotrophomonas bentonitica* into Se0 nanostructures and volatiles se species. Environ. Sci. Nano 7, 2140–2155. doi: 10.1039/D0EN00507J

[ref183] Ruiz-Fresneda M. A. Fernández-Cantos M. Gómez-Bolívar J. Eswayah A. S. Gardiner P. H. E. Pinel-Cabello M.. (2023). Combined bioreduction and volatilization of Se^VI^ by *Stenotrophomonas bentonitica*: formation of trigonal selenium nanorods and methylated species. Sci. Total Environ. 858:160030. doi: 10.1016/j.scitotenv.2022.160030, PMID: 36356742

[ref184] Ruiz-Fresneda M. A. Gomez-Bolivar J. Delgado-Martin J. del Mar Abad-Ortega M. Guerra-Tschuschke I. Merroun M. L. (2019). The bioreduction of selenite under anaerobic and alkaline conditions analogous to those expected for a deep geological repository system. Molecules 24:3868. doi: 10.3390/molecules24213868, PMID: 31717840 PMC6865132

[ref185] Ruiz-Fresneda M. A. Lopez-Fernandez M. Martinez-Moreno M. F. Cherkouk A. Ju-Nam Y. Ojeda J. J.. (2020b). Molecular binding of EuIII/CmIIIby *Stenotrophomonas bentonitica* and its impact on the safety of future Geodisposal of radioactive waste. Environ. Sci. Technol. 54, 15180–15190. doi: 10.1021/acs.est.0c02418, PMID: 33185105

[ref186] Sánchez-Castro I. Martínez-Rodríguez P. Abad M. M. Descostes M. Merroun M. L. (2021). Uranium removal from complex mining waters by alginate beads doped with cells of *Stenotrophomonas* sp. Br8: novel perspectives for metal bioremediation. J. Environ. Manage. 296:113411. doi: 10.1016/j.jenvman.2021.113411, PMID: 34351286

[ref187] Sánchez-Castro I. Martínez-Rodríguez P. Jroundi F. Solari P. L. Descostes M. Merroun M. L. (2020). High-efficient microbial immobilization of solved U^VI^ by the *Stenotrophomonas* strain Br8. Water Res. 183:116110. doi: 10.1016/j.watres.2020.116110, PMID: 32659540

[ref188] Sarikhani M. R. Malboobi M. Aliasgharzad N. Greiner R. (2019). Identification of two novel bacterial phosphatase-encoding genes in *pseudomonas putida* strain P13. J. Appl. Microbiol. 127, 1113–1124. doi: 10.1111/jam.14376, PMID: 31287935

[ref189] Sepulveda-Medina P. Katsenovich Y. Musaramthota V. Lee M. Lee B. Dua R.. (2015). The effect of uranium on bacterial viability and cell surface morphology using atomic force microscopy in the presence of bicarbonate ions. Res. Microbiol. 166, 419–427. doi: 10.1016/j.resmic.2015.03.003 25842164

[ref190] Shrestha R. Cerna K. Spanek R. Bartak D. Cernousek T. Sevcu A. (2022). The effect of low-pH concrete on microbial community development in bentonite suspensions as a model for microbial activity prediction in future nuclear waste repository. Sci. Total Environ. 808:151861. doi: 10.1016/j.scitotenv.2021.151861, PMID: 34838551

[ref191] Shrestha R. Černoušek T. Stoulil J. Kovářová H. Sihelská K. Špánek R.. (2021). Anaerobic microbial corrosion of carbon steel under conditions relevant for deep geological repository of nuclear waste. Sci. Total Environ. 800:149539. doi: 10.1016/j.scitotenv.2021.149539, PMID: 34392220

[ref192] Shukla A. Parmar P. Saraf M. (2017). Radiation, radionuclides and bacteria: An in-perspective review. J. Environ. Radioact. 180, 27–35. doi: 10.1016/j.jenvrad.2017.09.013 29024816

[ref193] Sivaswamy V. Boyanov M. I. Peyton B. M. Viamajala S. Gerlach R. Apel W. A.. (2011). Multiple mechanisms of uranium immobilization by *Cellulomonas* sp. strain ES6. Biotechnol. Bioeng. 108, 264–276. doi: 10.1002/bit.22956, PMID: 20872821

[ref194] Smart N. R. Reddy B. Rance A. P. Nixon D. J. Frutschi M. Bernier-Latmani R.. (2017). The anaerobic corrosion of carbon steel in compacted bentonite exposed to natural Opalinus clay porewater containing native microbial populations. Corros. Engineer. Sci. Technol. 52, 101–112. doi: 10.1080/1478422X.2017.1315233

[ref195] Smits K. M. Sakaki T. Howington S. E. Peters J. F. Illangasekare T. H. (2013). Temperature dependence of thermal properties of sands across a wide range of temperatures (30-70°C). Vadose Zone J. 138, 2256–2265. doi: 10.2136/vzj2012.0033

[ref196] Song D. Li X. Cheng Y. Xiao X. Lu Z. Wang Y.. (2017). Aerobic biogenesis of selenium nanoparticles by Enterobacter cloacae Z0206 as a consequence of fumarate reductase mediated selenite reduction. Sci. Rep. 7:3239. doi: 10.1038/s41598-017-03558-3, PMID: 28607388 PMC5468319

[ref197] Sreedevi P. R. Suresh K. Jiang G. (2022). Bacterial bioremediation of heavy metals in wastewater: a review of processes and applications. J. Water Process Engineer. 48:102884. doi: 10.1016/j.jwpe.2022.102884

[ref198] Staicu L. C. Wójtowicz P. J. Molnár Z. Ruiz-Agudo E. Gallego J. L. R. Baragaño D.. (2022). Interplay between arsenic and selenium biomineralization in Shewanella sp. O23S. Environ. Pollut. 306:119451. doi: 10.1016/j.envpol.2022.119451, PMID: 35569621

[ref199] Stylo M. Neubert N. Roebbert Y. Weyer S. Bernier-Latmani R. (2015). Mechanism of uranium reduction and immobilization in *Desulfovibrio vulgaris* biofilms. Environ. Sci. Technol. 49, 10553–10561. doi: 10.1021/acs.est.5b01769, PMID: 26251962

[ref200] Sun W. Nešic S. (2007). A mechanistic model of H2S corrosion of mild steel. In: *Paper presented at the CORROSION 2007*, Nashville, Tennessee, march 2007.

[ref201] Suriya J. Chandra Shekar M. Nathani N. M. Suganya T. Bharathiraja S. Krishnan M. (2017). Assessment of bacterial community composition in response to uranium levels in sediment samples of sacred Cauvery River. Appl. Microbiol. Biotechnol. 101, 831–841. doi: 10.1007/s00253-016-7945-2, PMID: 27812801

[ref202] Sutcliffe B. Chariton A. A. Harford A. J. Hose G. C. Greenfield P. Elbourne L. D. H.. (2017). Effects of uranium concentration on microbial community structure and functional potential. Environ. Microbiol. 19, 3323–3341. doi: 10.1111/1462-2920.13839, PMID: 28631400

[ref203] Suzuki Y. Kelly S. D. Kemner K. M. Banfield J. F. (2003). Microbial populations stimulated for hexavalent uranium reduction in uranium mine sediment. Appl. Microbiol. Biotechnol. 69, 1337–1346. doi: 10.1128/AEM.69.3.1337-1346.2003 PMC150047 12620814

[ref204] Tabak H. H. Lens P. van Hullebusch E. D. Dejonghe W. (2005). Developments in bioremediation of soils and sediments polluted with metals and radionuclides-1. Microbial processes and mechanisms affecting bioremediation of metal contamination and influencing metal toxicity and transport. Rev. Environ. Sci. Biotechnol. 4, 115–156. doi: 10.1007/s11157-005-2169-4

[ref205] Tan Y. Wang Y. Wang Y. Xu D. Huang Y. Wang D.. (2018). Novel mechanisms of selenate and selenite reduction in the obligate aerobic bacterium *Comamonas testosteroni* S44. J. Hazard. Mater. 359, 129–138. doi: 10.1016/j.jhazmat.2018.07.014, PMID: 30014908

[ref206] Tan Y. Yao R. Wang R. Wang D. Wang G. Zheng S. (2016). Reduction of selenite to se(0) nanoparticles by filamentous bacterium *Streptomyces* sp. ES2-5 isolated from a selenium mining soil. Microb. Cell Fact. 15:157. doi: 10.1186/s12934-016-0554-z, PMID: 27630128 PMC5024524

[ref207] Theodorakopoulos N. Chapon V. Coppin F. Floriani M. Vercouter T. Sergeant C.. (2015). Use of combined microscopic and spectroscopic techniques to reveal interactions between uranium and *microbacterium* sp. A9, a strain isolated from the Chernobyl exclusion zone. J. Hazard. Mater. 285, 285–293. doi: 10.1016/j.jhazmat.2014.12.018, PMID: 25528226

[ref208] Tišáková L. Pipíška M. Godány A. Horník M. Vidová B. Augustín J. (2013). Bioaccumulation of 137Cs and 60Co by bacteria isolated from spent nuclear fuel pools. J. Radioanal. Nucl. Chem. 295, 737–748. doi: 10.1007/s10967-012-1932-6

[ref209] Tripathy S. Thomas H. R. Stratos P. (2017). Response of compacted bentonites to thermal and thermo-hydraulic loadings at high temperatures. Geosciences 7:53. doi: 10.3390/geosciences7030053

[ref210] Ullah A. Yin X. Wang F. Xu B. Mirani Z. A. Xu B.. (2021). Biosynthesis of selenium nanoparticles (via Bacillus subtilis BSN313), and their isolation, characterization, and bioactivities. Molecules 26:5559. doi: 10.3390/molecules26185559, PMID: 34577029 PMC8468162

[ref211] UNSCEAR. (2013), Sources, effects and risks of ionizing radiation: report to the general assembly with scientific annexes. UNSCEAR 2013 report volume I. New York, United Nations, 2014. Available at: http://www.unscear.org/unscear/en/publications/2013_1.html

[ref212] Videla H. A. Herrera L. K. (2009). Understanding microbial inhibition of corrosion. A comprehensive overview. Int. Biodeter. Biodegr. 63, 896–900. doi: 10.1016/j.ibiod.2009.02.002

[ref213] Villar M. V. Pérez del Villar L. Martín P. L. Pelayo M. Fernández A. M. Garralon A.. (2006). The study of Spanish clays for their use as sealing materials in nuclear waste repositories: 20 years of progress. J. Iber. Geol. 32, 15–36.

[ref214] Vogel M. Fischer S. Maffert A. Hübner R. Scheinost A. C. Franzen C.. (2018). Biotransformation and detoxification of selenite by microbial biogenesis of selenium-sulfur nanoparticles. J. Hazard. Mater. 344, 749–757. doi: 10.1016/j.jhazmat.2017.10.034, PMID: 29156387

[ref215] Waite T. D. Davis J. A. Payne T. E. Waychunas G. A. Xu N. (1994). Uranium^VI^ adsorption to ferrihydrite: application of a surface complexation model. Geochim. Cosmochim. Acta 58, 5465–5478. doi: 10.1016/0016-7037(94)90243-7

[ref216] Wang M. Jiang D. Huang X. (2022). Selenium nanoparticle rapidly synthesized by a novel highly selenite-tolerant strain *Proteus penneri* LAB-1. iScience 25:104904. doi: 10.1016/j.isci.2022.104904, PMID: 36097619 PMC9463581

[ref217] Wang Y. Shu X. Zhou Q. Fan T. Wang T. Chen X.. (2018). Selenite reduction and the biogenesis of selenium nanoparticles by *Alcaligenes faecalis* se03 isolated from the gut of *Monochamus alternatus* (Coleoptera: Cerambycidae). Int. J. Mol. Sci. 19:2799. doi: 10.3390/ijms19092799, PMID: 30227664 PMC6164237

[ref219] Xu Y. Zeng Z. Lv H. (2019). Temperature dependence of apparent thermal conductivity of compacted bentonites as buffer material for high-level radioactive waste repository. Appl. Clay Sci. 174, 10–14. doi: 10.1016/j.clay.2019.03.017

[ref220] Xu C. Zhang Y. Cheng G. Zhu W. (2008). Pitting corrosion behavior of 316L stainless steel in the media of sulfate-reducing and iron-oxidizing bacteria. Mater Charact 59, 245–255. doi: 10.1016/j.matchar.2007.01.001

[ref221] Yang T. Chen M. L. Wang J. H. (2015). Genetic and chemical modification of cells for selective separation and analysis of heavy metals of biological or environmental significance. Trends Anal. Chem. 66, 90–102. doi: 10.1016/j.trac.2014.11.016, PMID: 36852718

[ref222] You W. Peng W. Tian Z. Zheng M. (2021). Uranium bioremediation with U^VI^-reducing bacteria. Sci. Total Environ. 798:149107. doi: 10.1016/j.scitotenv.2021.149107, PMID: 34325147

[ref223] Yun J. Malvankar N. S. Ueki T. Lovley D. R. (2016). Functional environmental proteomics: elucidating the role of a c-type cytochrome abundant during uranium bioremediation. ISME J. 10, 310–320. doi: 10.1038/ismej.2015.113, PMID: 26140532 PMC4737924

[ref224] Yung M. C. Jiao Y. (2014). Biomineralization of uranium by PhoY phosphatase activity aids cell survival in Caulobacter crescentus. Appl. Environ. Microbiol. 80, 4795–4804. doi: 10.1128/AEM.01050-14, PMID: 24878600 PMC4135761

[ref225] Zambonino M. C. Quizhpe E. M. Jaramillo F. E. Rahman A. Santiago Vispo N. Jeffryes C.. (2021). Green synthesis of selenium and tellurium nanoparticles: current trends, biological properties and biomedical applications. Int. J. Mol. Sci. 22:989. doi: 10.3390/ijms22030989, PMID: 33498184 PMC7863925

[ref226] Zhang X. Gu P. Liu Y. (2019). Decontamination of radioactive wastewater: state of the art and challenges forward. Chemosphere 215, 543–553. doi: 10.1016/j.chemosphere.2018.10.029, PMID: 30342399

[ref227] Zhang J. Song H. Chen Z. Liu S. Wei Y. Huang J.. (2018). Biomineralization mechanism of U(VI) induced by *Bacillus cereus* 12-2: the role of functional groups and enzymes. Chemosphere 206, 682–692. doi: 10.1016/j.chemosphere.2018.04.181, PMID: 29783053

[ref228] Zheng L. Rutqvist J. Xu H. Birkholzer J. T. (2017). Couple THMC models for bentonite in an argillite repository for nuclear waste: Illitization and its effect on swelling stress under high temperature. Eng. Geol. 230, 118–129. doi: 10.1016/j.enggeo.2017.10.002

[ref229] Zhong J. Hu X. Liu X. Cui X. Lv Y. Tang C.. (2021). Isolation and identification of uranium tolerant phosphate-solubilizing bacillus spp. and their synergistic strategies to U(VI) immobilization. Front. Microbiol. 12:676391. doi: 10.3389/fmicb.2021.676391, PMID: 34326819 PMC8313988

